# Storage lipid studies in tuberculosis reveal that foam cell biogenesis is disease-specific

**DOI:** 10.1371/journal.ppat.1007223

**Published:** 2018-08-30

**Authors:** Valentina Guerrini, Brendan Prideaux, Landry Blanc, Natalie Bruiners, Riccardo Arrigucci, Sukhwinder Singh, Hsin Pin Ho-Liang, Hugh Salamon, Pei-Yu Chen, Karim Lakehal, Selvakumar Subbian, Paul O’Brien, Laura E. Via, Clifton E. Barry, Véronique Dartois, Maria Laura Gennaro

**Affiliations:** 1 Public Health Research Institute, New Jersey Medical School, Rutgers, The State University of New Jersey, Newark, NJ, United States of America; 2 Department of Pathology and Laboratory Medicine, New Jersey Medical School, Rutgers, The State University of New Jersey, Newark, NJ, United States of America; 3 Knowledge Synthesis, Berkeley, CA, United States of America; 4 Tuberculosis Research Section, National Institute of Allergy and Infectious Diseases, National Institutes of Health, Bethesda, MD, United States of America; University of Massachusetts Medical School, UNITED STATES

## Abstract

Foam cells are lipid-laden macrophages that contribute to the inflammation and tissue damage associated with many chronic inflammatory disorders. Although foam cell biogenesis has been extensively studied in atherosclerosis, how these cells form during a chronic infectious disease such as tuberculosis is unknown. Here we report that, unlike the cholesterol-laden cells of atherosclerosis, foam cells in tuberculous lung lesions accumulate triglycerides. Consequently, the biogenesis of foam cells varies with the underlying disease. In vitro mechanistic studies showed that triglyceride accumulation in human macrophages infected with *Mycobacterium tuberculosis* is mediated by TNF receptor signaling through downstream activation of the caspase cascade and the mammalian target of rapamycin complex 1 (mTORC1). These features are distinct from the known biogenesis of atherogenic foam cells and establish a new paradigm for non-atherogenic foam cell formation. Moreover, they reveal novel targets for disease-specific pharmacological interventions against maladaptive macrophage responses.

## Author summary

The formation of foam cells (lipid-laden macrophages) is a maladaptive host response associated with chronic inflammation. Foam cell biogenesis has been most thoroughly studied in atherosclerosis, where it is linked to disruption of cholesterol homeostasis and consequent intracellular accumulation of cholesteryl esters. In this study, we show that, during pulmonary tuberculosis, foam cells found in necrotizing granulomas (tubercles) accumulate predominantly triglycerides rather than cholesteryl esters. Triglyceride profiles are highly conserved across lung granulomas in rabbits, non-human primates, and humans. We also show that triglyceride accumulation in human primary macrophages infected with *Mycobacterium tuberculosis* involves TNF receptor signaling and downstream activation of mTORC1 and caspase pathways. Our finding that tuberculous foam cells differ from atherogenic foam cells with respect to storage lipid composition and lipid accumulation mechanism reveals that foam cell formation is a disease-specific process. The results of this study point to novel targets for pharmacological intervention against tuberculosis and help explain links between tuberculosis and insulin resistance.

## Introduction

Formation of foam cells (lipid-laden macrophages) is a manifestation of maladaptive responses occurring during chronic inflammatory conditions [1]. The best-studied case is that of atherosclerosis. There, retention of lipoproteins in the arterial intima triggers extravasation of circulating monocytes and subsequent accumulation of lipids, predominantly cholesteryl esters, in the cytoplasm of monocyte-derived macrophages. The resulting foam cells exhibit impaired immune functions; they also produce pro-inflammatory mediators and release cellular content upon death [2]. By maintaining inflammation and failing to resolve it, foam cells contribute to a chronic inflammatory state and consequently to tissue damage [2, 3]. Foam cells are observed well beyond the hyperlipidemia associated with atherosclerosis: these lipid-laden macrophages are found in many diseases of non-infectious (e.g., autoimmune) and infectious (e.g., HIV, tuberculosis) etiology, where foam cells are often central to pathogenesis [4]. Since these diseases are associated with chronic inflammation rather than hyperlipidemia, the mechanisms triggering foam cell formation in these pathologies may differ from those occurring during atherosclerosis. Whether that is the case is currently unknown.

Foam cells are among the macrophages located in granulomas, the multi-cellular aggregates found in tuberculosis, an infectious disease caused by the intracellular pathogen *Mycobacterium tuberculosis* [5, 6]. The presence of foam cells is typically associated with necrotic granulomas, where these lipid-laden macrophages are located in the innermost cellular layers and also surround the necrotic material (caseum) at the center of the lesion [6, 7]. Indeed, death of foam cells and release of their contents likely contribute to sustained inflammation and the formation of caseum. Granuloma caseation and enlargement lead to progressive destruction of lung tissue and loss of pulmonary function [8, 9]. The caseum also contains the extracellular tubercle bacilli that are eventually released into the external environment when granulomas cavitate [6]. Despite the important contributions of foam cells to tuberculosis pathogenesis, disease progression, and transmission of infection, the events involved in tuberculous foam cell formation remain unknown.

The key event in foam cell biogenesis is the intracellular accumulation of lipid droplets. These are cytoplasmic quasi-organelles that consist of storage neutral lipids, primarily cholesteryl esters (CE) and/or triglycerides (TAG), surrounded by a monolayer of phospholipids containing structural proteins, trace free cholesterol, and enzymes [10–12]. The relative abundance of TAG and CE in lipid droplets varies with cell type [10], suggesting specialized functions. For example, in white adipocytes, which are highly adapted for lipid storage, lipid droplets are almost exclusively TAG. In contrast, in steroidogenic cells, which are the sites of steroid hormone biosynthesis, lipid droplets are enriched in CE. Lipid droplets induced by infection also differ in lipid composition depending on the host cell type and the pathogen [12]. For example, hepatocytes chronically infected with hepatitis C virus accumulate TAG [13], while Schwann cells infected with *Mycobacterium leprae* contain increased levels of cholesterol and CE [14]. Macrophages change their physiology in response to particular environmental cues [15]: in vitro they can accumulate either TAG- or CE-rich droplets depending on the stress condition [16, 17]. Studies of macrophages during disease have focused largely on cholesterol accumulation in atherogenic foam cells—understanding lipid content and mechanisms of foam cell formation in non-atherogenic diseases requires new, disease-specific studies.

Here we report that foam cells in tuberculous lung granulomas are enriched in TAG. The TAG species in caseum and foam-cell-rich areas of tuberculous granulomas are remarkably conserved across humans and two animal species commonly used to model necrotizing granulomas. We also show that TAG accumulation in human primary macrophages infected in vitro with *M*. *tuberculosis* requires Tumor Necrosis Factor Receptor (TNFR) signaling and the downstream activation of the caspase cascade and of the mammalian target of rapamycin complex 1 (mTORC1). Since these pro-adipogenic mechanisms differ radically from those known to occur during atherogenesis, our findings show that foam cell biogenesis is a disease-specific process. In addition, since mTORC1 and TNFα contribute to insulin resistance [18–21], the involvement of these two factors in tuberculosis pathogenesis provide a mechanistic explanation for the association between tuberculosis and insulin resistance.

## Results

### Triglycerides are the dominant storage lipids in the tuberculous granuloma

**Fig 1:**
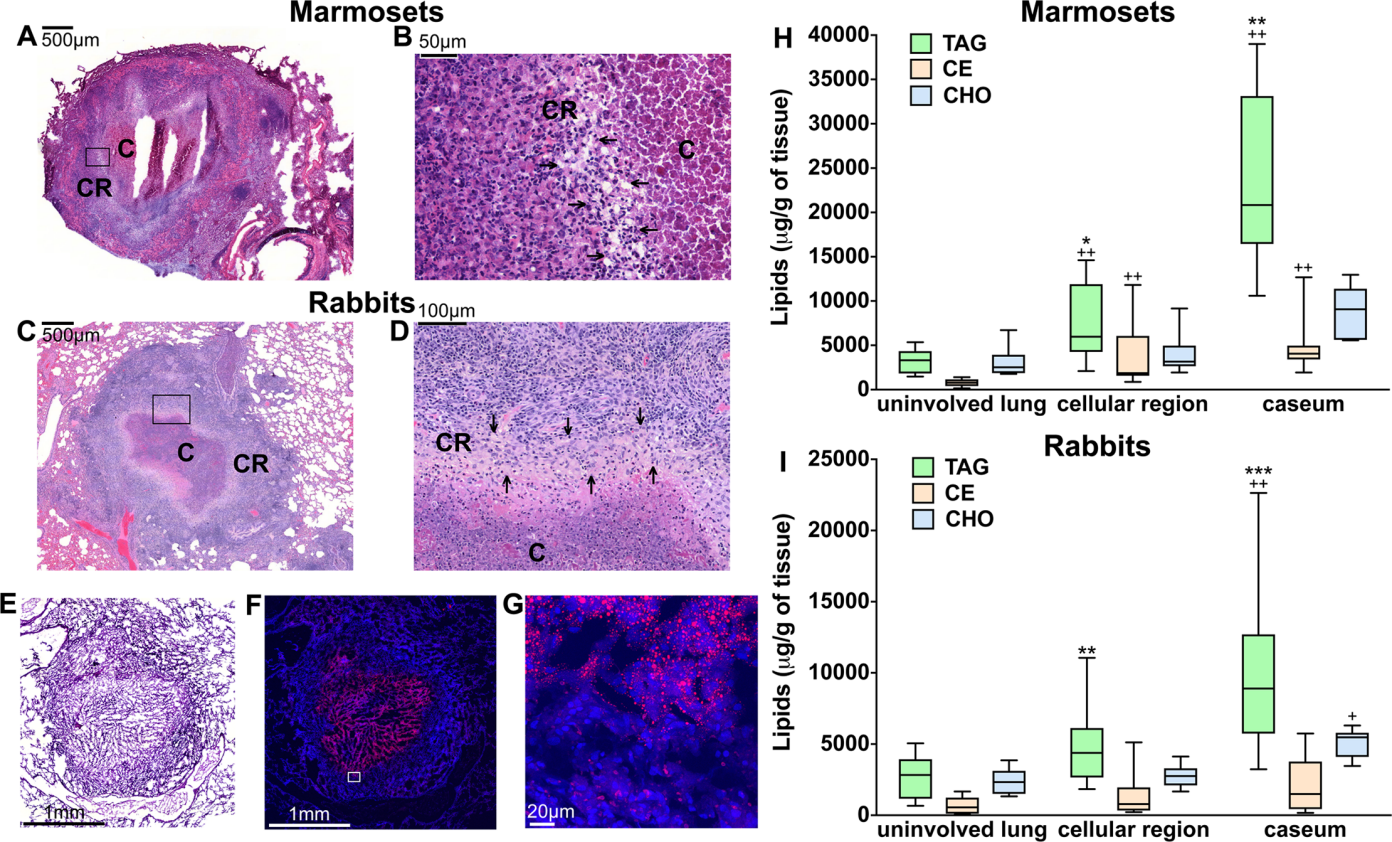
TAG, CE, and free cholesterol levels in pulmonary tuberculous lesions of marmosets and rabbits. Haematoxylin and eosin staining of lung tissue sections from *M*. *tuberculosis*-infected marmosets (**A-B**) and rabbits (**C-D**). In panels **A** and **C**, the boxes indicate the regions of granulomas that are shown at higher magnification in panels **B** and **D**. In necrotizing granulomas, the center of the lesion is occupied by caseum (C) (“cheese-like” material that becomes increasingly acellular as necrosis progresses), which is surrounded by a cellular region (CR) containing macrophages and lymphocytes. The inner layers of the cellular region are enriched in macrophages, including foam cells, epithelioid, and multinucleated giant cells; lymphocytes are predominantly found in the outermost cellular area [6, 7, 9]. In panels **B** and **D**, foam-cell rich regions are indicated by arrows. The presence of foam cells is demonstrated by the vacuole-rich areas resulting from loss of lipids during tissue preparation for haematoxylin and eosin staining. (**E**) Haematoxylin and eosin staining of the *M*. *tuberculosis*-infected rabbit lung tissue section used for confocal microscopy. The haematoxylin and eosin staining differs in intensity from the images in panels **A** through **D** because it was performed after fluorescence staining, to confirm the structure of the lesion. **(F-G)** Confocal microscopy images of tuberculous rabbit lung tissue sections stained with Nile red (red) and DAPI (blue). In Panel **F,** the box indicates the region of the granuloma that is shown at higher magnification in panel **G.** Lipid droplets stained with Nile Red, a dye widely used to visualize lipid droplet-laden cells both in vitro and in vivo [7, 71, 85, 105], are visible in the cellular region surrounding the caseum. A three-dimensional image of foam cells is provided in the S1 Video. (**H-I**) Measurements of TAG, CE, and free cholesterol levels. Areas of caseum and macrophage-rich cellular region of lung lesions, and regions of uninvolved lung were sampled by laser capture microdissection from 4–10 rabbits and 6–9 marmosets (1–2 samples per animal). Lipids were extracted and TAG, CE species, and free cholesterol quantified by LC-MS. All measurements are expressed as micrograms of lipid per gram of tissue (μg/g). The box plots show lower quartile, median, and upper quartile of the distribution. The whiskers represent the minimum and maximum values. Statistical significance (*p* < 0.025) was calculated by the Mann-Whitney and Wilcoxon signed-rank tests and the multiple-comparison Bonferroni correction. Statistical differences between lesional and uninvolved lung areas (^+^*p* < 0.025, ^++^*p* < 0.01 by the Mann-Whitney test), and between TAG and CE (*p <0.025, **p < 0.01, ***p < 0.001 by the Wilcoxon signed-rank test) are indicated. TAG: triglycerides, CE: cholesteryl esters, CHO: free cholesterol.

Since storage lipids in foam cells are primarily CE and/or TAG, we measured the abudance of these two lipid classes in tuberculous lung lesions. We also measured levels of free cholesterol since this lipid has been implicated in tuberculosis pathogenesis (reviewed in [8]). For these analyses, we used rabbits and marmosets (a small, New World monkey) since, when experimentally infected with *M*. *tuberculosis*, these animal species develop necrotizing granulomas similar to those found in human tuberculosis [22, 23] (Fig 1A, 1B, 1C and 1D). Three lung regions were separately sampled by laser capture microdissection: the caseum and surrounding cellular region of granulomas, and the uninvolved lung (see Fig 1A, 1B, 1C and 1D for haematoxylin and eosin staining, Fig 1F and 1G and S1 Video for Nile red staining, and S1 Fig for laser capture microdissection). TAG and CE species and free cholesterol were individually quantified in the microdissected samples by liquid chromatography-mass spectrometry (LC-MS) (lipid standards are listed in S1 Table). Total TAG, CE, and free cholesterol are shown in Fig 1H and 1I. We found that the amount of TAG was higher in the caseum and cellular regions of the lesions relative to the uninvolved lung tissue in both animal species (up to 6-fold) (Fig 1H and 1I). In contrast, only in marmosets was the CE content elevated in the lesions relative to uninvolved tissue (Fig 1H), while the amount of free cholesterol changed little, if at all, in the lesional versus uninvolved tissue in both animal species (only the rabbit caseum showed borderline significance relative to uninvolved tissue). Notably, the amount of TAG always exceeded that of CE (up to 6-fold) in the lesions of both species (Fig 1H and 1I). Collectively, the data showed that TAGs are the predominant storage lipids in the necrotic core and foam-cell-rich areas of tuberculous granulomas.

### Triglyceride profiles in tuberculous necrotic lesions are conserved in animal models and in human lung

In principle it is desirable to assess the characteristics of tuberculous lesions in humans. However, when we quantified the storage lipids in microdissected caseum and surrounding cellular areas of lung lesions from two multidrug-resistant tuberculosis patients (see S2A, S2B and S2C Fig for histopathological and drug treatment information), we found that the relative abundance of CE species varied greatly between the two donors (S2D Fig for total TAG and CE content; S2 and S3 Tables for individual TAG and CE species). Thus, no conclusion could be drawn in terms of predominance of one storage lipid over the other, due to small sample size, the potential confounding effects of prolonged lung disease and antibiotic treatment, and/or additional donor-specific variables.

**Fig 2:**
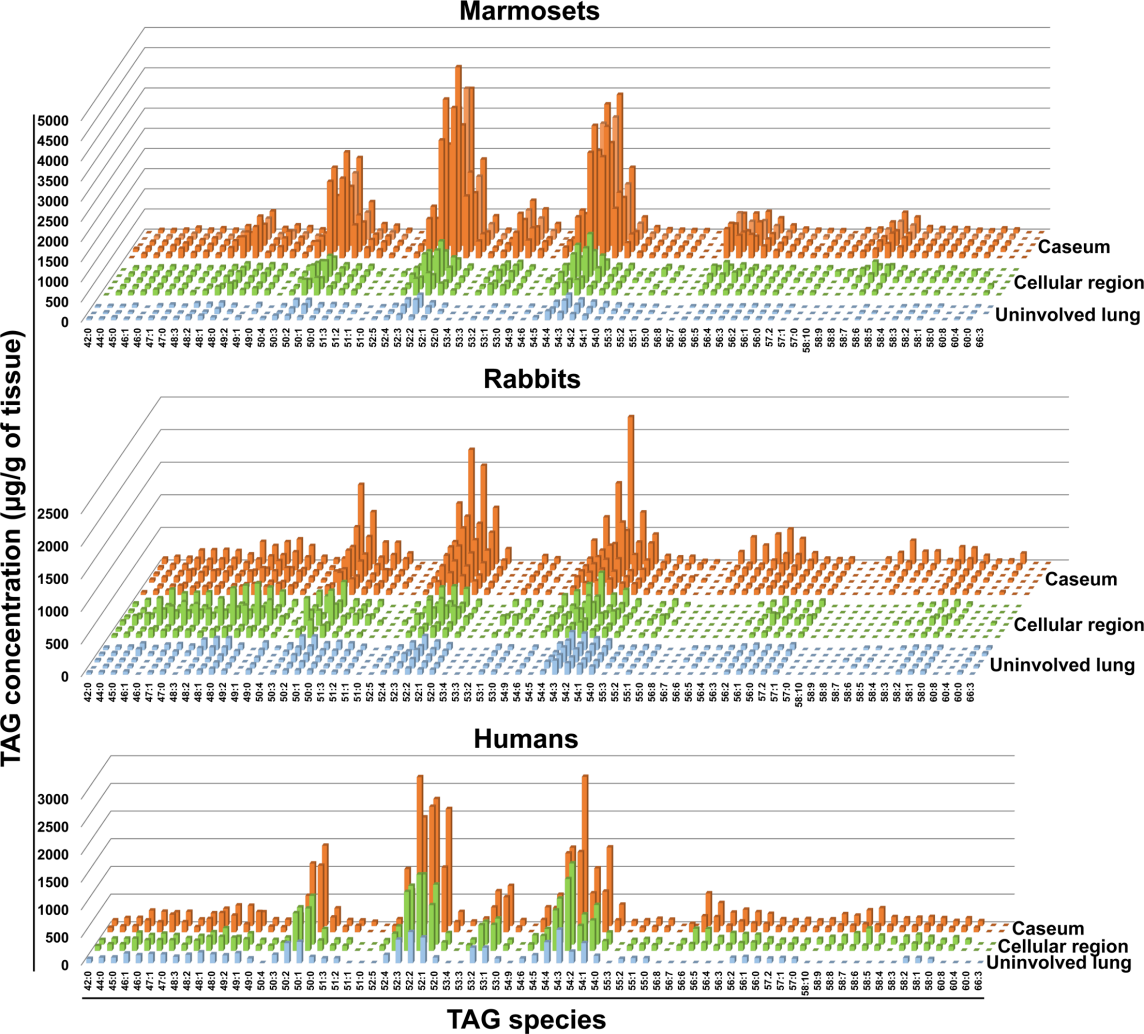
TAG species profiles in pulmonary tuberculous lesions of rabbits, marmosets, and humans. TAG species in microdissected areas of caseum and cellular regions of lesions, and uninvolved lung areas were quantified by LC-MS. In all graphs, each line represents one sample: uninvolved lung areas (light blue), cellular region (green), and caseum (orange). Marmosets: five lesional and three uninvolved lung areas (from four animals). Rabbits: six lesional and five uninvolved lung areas (from six animals). Humans: two lesional areas (one per patient) from two active tuberculosis cases, and one uninvolved lung area (from one of the two patients). One of the dominant species in this figure (TAG 52:2) was revealed by the electrospray ionization mass spectrometry analysis of human tuberculous caseum in a previous study [85].

We were surprised, however, to observe that the TAG profiles seen in rabbit, marmoset, and human lesions were highly conserved (Fig 2). Three clusters were most abundant: TAG containing fatty acid chains of (i) 50 carbons and 0–3 double bonds, (ii) 52 carbons and 0–4 double bonds, and (iii) 54 carbons and 1–5 double bonds. The most abundant species in each cluster were also conserved (S2 Table). In contrast, when we analyzed the CE profiles, only two species were prominent in the human samples and much more variability was observed within and across the animal species (S3 Table). These results provide robust evidence that a multi-species TAG profile is conserved in tuberculous necrotizing granulomas, regardless of host species.

### Human macrophages infected in vitro with *M*. *tuberculosis* accumulate triglyceride-rich lipid droplets

**Fig 3:**
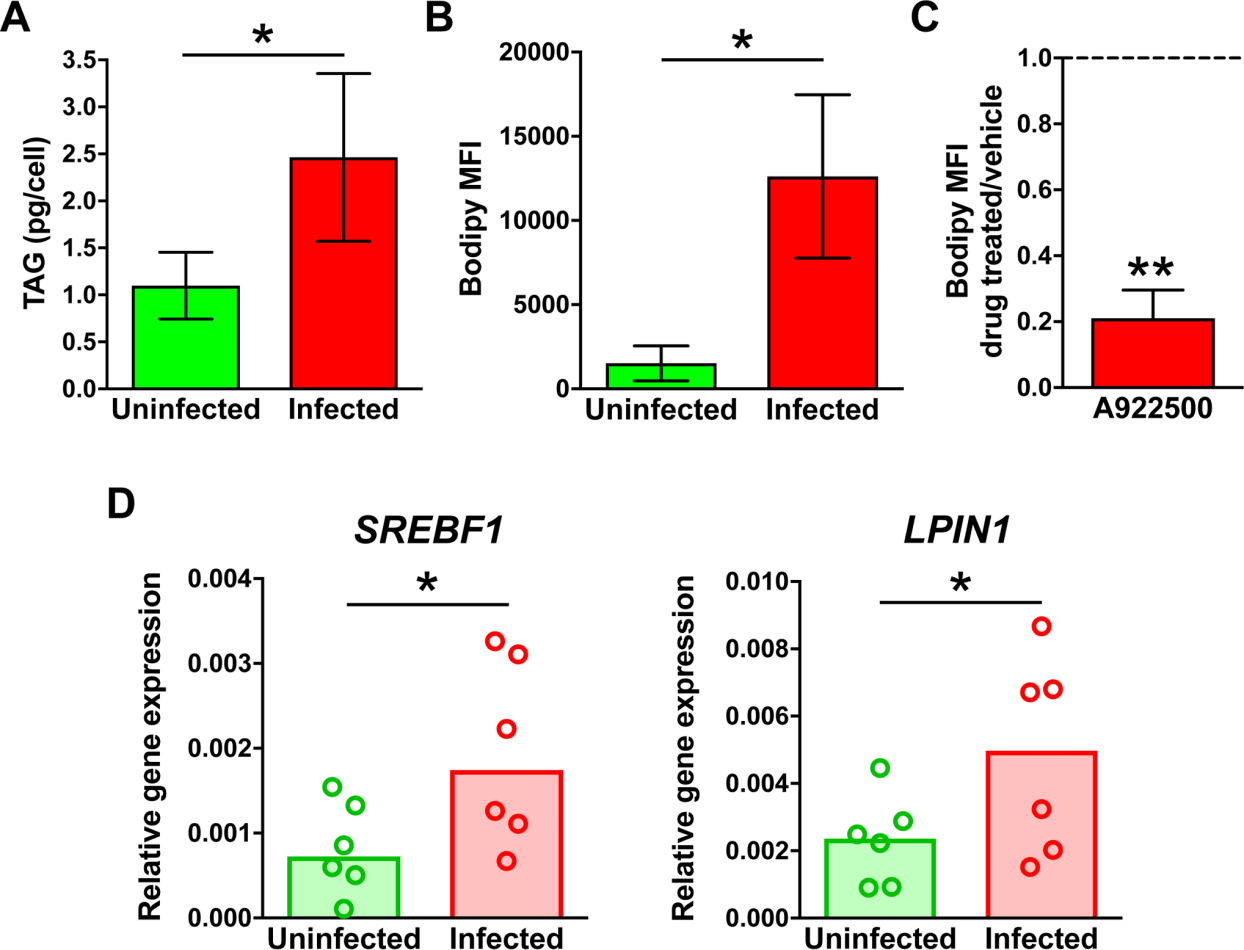
TAG and lipid droplet levels, and expression of TAG metabolism genes in macrophages infected with *M*. *tuberculosis* in vitro. Human monocyte-derived macrophages (MDM) were infected with *M*. *tuberculosis* for 24 h or left uninfected. Infected cells were treated with either DMSO (vehicle control) or A922500 (DGAT inhibitor). (**A**) TAG measurement by mass spectrometry. Lipids were extracted from uninfected and infected cells, and TAG species quantified by LC-MS. (**B**) Lipid droplet content determination by imaging flow cytometry. Uninfected and infected macrophages were stained with Bodipy 493/503 and imaged by ImageStream^X^Mark II imaging flow cytometer at 60× magnification. Images were analyzed by IDEAS software and lipid droplet content was expressed as median Bodipy fluorescence intensity per cell (MFI) (the baseline measurements in uninfected cells reflect the scanty lipid droplet induction occurring during macrophage differentiation in vitro). (**C**) Effect of A922500 on lipid droplet content. Lipid droplet content of infected macrophages was determined as described. Results were expressed as ratio between inhibitor- and vehicle-treated cells. In **A**, **B**, and **C**, average and standard deviation of three donors are shown. (**D**) Gene expression analysis. RNA was isolated from uninfected and infected cells, and mRNAs enumerated by qPCR using gene-specific primers and molecular beacons (S5 Table). Gene expression was calculated using the 2 ^-ΔΔCt^ method and normalized to the housekeeping *ACTB* gene. Graphs show the medians of six donors, with each dot representing one donor. Statistical significance (*p < 0.05, **p < 0.01) was assessed by paired (panels **A**, **B**, and **D**) and one-sample (panel **C**) student t-tests. The comparisons in the paired tests are as indicated; the comparison in the one-sample student t-test was between treated and untreated cells. *SREBF1* encodes SREBP-1c, a transcription factor that functions as master regulator of TAG biosynthesis [106]; *LPIN1* encodes LIPIN1, a TAG biosynthetic enzyme [107] that also functions as transcriptional coactivator of SREBP-1c [108].

To characterize the underlying mechanism of tuberculosis-induced TAG accumulation, we examined human monocyte-derived macrophages (MDM) infected in vitro with *M*. *tuberculosis*. We first focused on the effect of infection on storage lipid content of these macrophages using LC-MS. We observed a 2.5-fold increase in TAG levels due to infection (Fig 3A). The TAG species profile (S2 Table) was similar to that observed in the necrotizing granulomas taken from patients (S3 Fig), and levels of CE were below the limit of quantification. The elevated TAG levels in infected cells were accompanied by higher lipid droplet content relative to that of uninfected cells (~8-fold), as detected by imaging flow cytometry (Fig 3B). Furthermore, lipid droplet accumulation was abolished when infected macrophages were treated with a chemical inhibitor (A922500) of the DGAT1 enzyme, which mediates the conversion of di- to tri-glycerides (Fig 3C). Collectively, these data show that TAG is the dominant lipid in lipid droplets formed in *M*. *tuberculosis-*infected macrophages. Consistent with the observed TAG accumulation, macrophage infection also increased expression of sentinel TAG biosynthesis genes, as determined by RT-PCR (Fig 3D).

In complementary experiments, we found no free cholesterol in lipid droplets isolated from the THP-1 macrophage-like human cell line (S4A, S4B and S4C Fig): free cholesterol was detected by LC-MS in whole cell lysates (membrane cholesterol) but not in the isolated lipid droplet material (S4D and S4E Fig). In addition, the increase in lipid droplet content caused by *M*. *tuberculosis* infection was accompanied by increased levels of TAG but not free cholesterol (S4F and S4G Fig). Moreover, treatment with BM 15766, an inhibitor of 7-dehydrocholesterol reductase (the enzyme catalyzing the last step of *de novo* cholesterol synthesis), failed to alter infection-induced lipid droplet accumulation (S4H Fig). Together, these results indicate that lipid droplets do not accumulate free cholesterol, as expected from lipid droplet biology [12].

### Triglyceride accumulation in *M*. *tuberculosis*-infected human macrophages requires TNFα signaling

**Fig 4:**
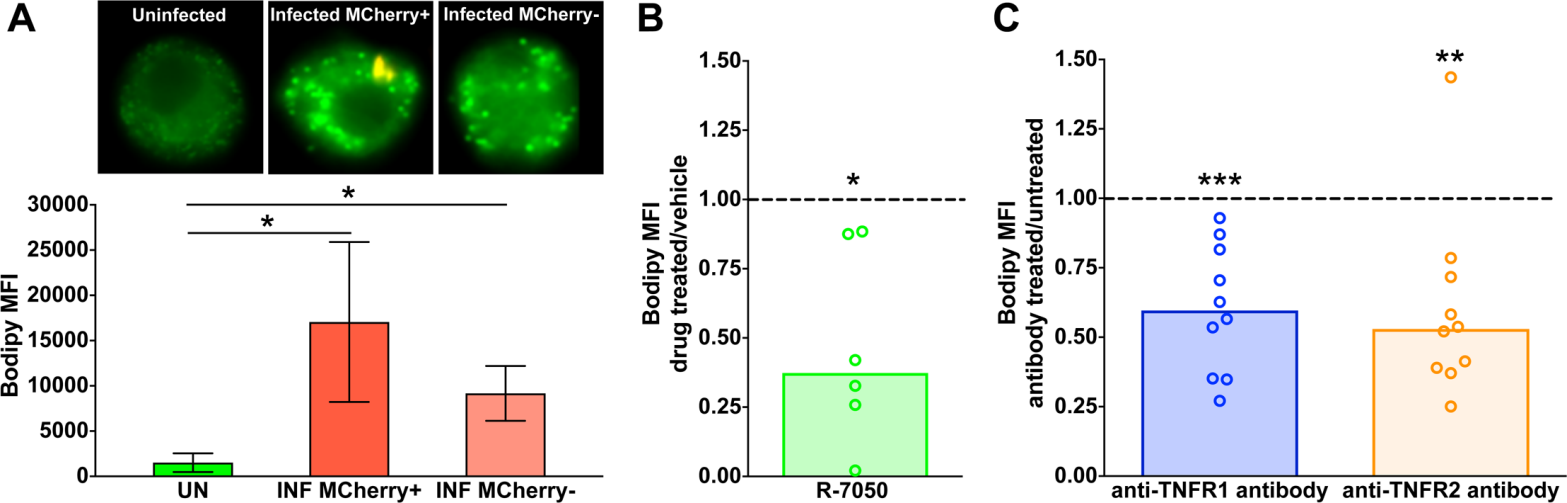
Role of TNFR in lipid droplet formation in *M*. *tuberculosis-*infected macrophages. MDM were infected with mCherry-expressing *M*. *tuberculosis* and treated either with R-7050 (TNFR chemical inhibitor) or DMSO (vehicle control). When TNFR neutralizing antibodies were used, MDM were either pre-treated with the antibodies or left untreated, and then infected. Uninfected samples were included as controls. Lipid droplet content was measured as described in Fig 3. (**A**) Representative images of uninfected and infected macrophages obtained by imaging flow cytometry (60× magnification) and lipid droplet content determination; lipid droplets are fluorescent green signals and bacteria are orange signals. Each bar of the graph represents the average and standard deviation of median Bodipy fluorescence intensity per cell obtained from three donors. Effect of R-7050 (**B**) or TNFR neutralizing antibodies (**C**) on lipid droplet content of infected macrophages. Results are expressed as the ratio between lipid droplet content of treated and untreated cells. Graphs show the median of six (**B**) and ten (**C**) donors (each dot represents one donor). Statistical significance (*p < 0.05, **p < 0.01, ***p < 0.001) was assessed by paired (panel **A**) and one-sample (panels **B** and **C**) student t-tests. The comparisons in the paired tests are as indicated; the comparison in the one-sample student t-test was between treated and untreated cells. UN: uninfected, INF: infected, TNFR1 AB: TNFR1 neutralizing antibodies, TNFR2 AB: TNFR2 neutralizing antibodies.

Based on the results described above, we used lipid droplet levels as a surrogate measure of TAG levels in MDM. Imaging flow cytometric analysis of lipid droplets in *M*. *tuberculosis*-infected macrophages makes it possible to co-localize lipid droplets (stained with the fluorescent dye Bodipy 493/503) with intracellular tubercle bacilli (expressing the fluorescent protein mCherry). Within the cultures of infected macrophages, macrophages that lacked intracellular bacilli (mCherry negative) also exhibited elevated lipid droplet content, albeit at a lower level than bacilli-bearing cells (mCherry positive) (the comparison with non-infected cells was statistically significant for both mCherry-positive and mCherry-negative cells) (Fig 4A). This observation indicated that a paracrine signal may be associated with infection-induced lipid droplet accumulation.

A potential candidate for paracrine signaling in tuberculosis is TNFα, a pro-inflammatory cytokine induced during *M*. *tuberculosis* infection [24], since this cytokine can alter cellular lipid homeostasis (it may induce either lipid accumulation or lipid degradation, depending on cell type and conditions; reviewed in [25–27]). To examine the involvement of TNFα in *M*. *tuberculosis*-induced TAG accumulation, infected macrophages were treated with a chemical inhibitor (R-7050) of the TNF receptor pathway. This treatment decreased lipid droplet content by 60% on average (Fig 4B). A similar lipid droplet reduction was observed when macrophages were treated with antibodies that block the function of either TNFα receptor (TNFR1 and TNFR2) and then infected with *M*. *tuberculosis* (Fig 4C). Thus, TAG accumulation requires TNFα signaling, with both receptors contributing to this process. The involvement of TNFα receptor is consistent with the expression of TNF receptor-associated factors reported in *M*. *tuberculosis*-induced foam cells [28]. The incomplete block of TAG accumulation can be explained as incomplete pharmacological inhibition, or by additional mechanisms of TAG accumulation during infection, or both.

### Triglyceride accumulation in *M*. *tuberculosis*-infected human macrophages requires mTORC1 signaling and caspase activation

**Fig 5:**
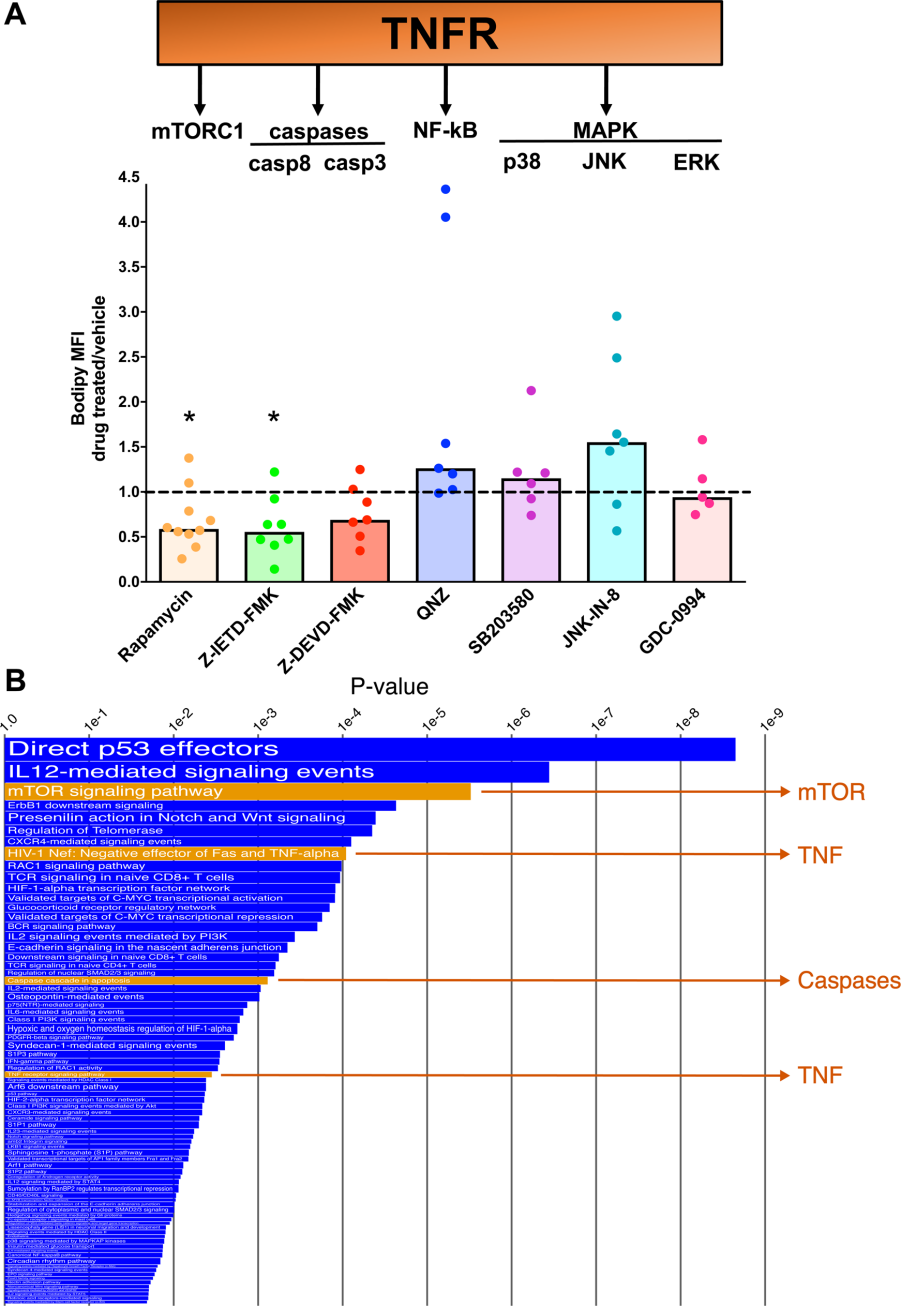
Role of TNFR-mediated pathways in lipid droplet formation in *M*. *tuberculosis-*infected macrophages. (**A**) Effect of pathway inhibitors on lipid droplet content of infected macrophages. The scheme at the top of the figure shows the intracellular signaling pathways activated by TNFR1 and TNFR2. Infected MDM were treated either with chemical inhibitors or vehicle; lipid droplet content was measured and results expressed as described in Fig 4. Statistical significance (*p < 0.05) of differences between treated and untreated cells was assessed by one-sample student t-test. Rapamycin: mTORC1 inhibitor, Z-IETD-FMK: caspase 8 inhibitor, Z-DEVD-FMK: caspase 3 inhibitor, QNZ: NF-κB inhibitor, SB203580: p38 inhibitor, JNK-IN-8: JNK inhibitor, GDC-0994: ERK inhibitor. (**B**) Transcriptomic analysis of human lung tuberculous granulomas. The differential expression between sample classes was determined for 182 Pathway Interaction Database pathways by coincident extreme ranks in numerical observations. 77 pathways were identified at a cutoff false discovery rate of 0.05; the *p* values for these were plotted onto the *x*-axis. To represent effect size, pathway gene sets with fewer genes were given greater bar height than were larger sets that yielded similar *p* values. Four of the pathways discussed in the text are highlighted in orange.

We next sought to identify cellular functions regulated by TNF signaling that are involved in TAG accumulation in *M*. *tuberculosis*-infected macrophages. Binding of TNFα to its receptors activates multiple signaling pathways that collectively regulate cellular metabolism, cell proliferation, cell survival, and inflammatory responses (Fig 5A) [27, 29]. These pathways include: (i) the mammalian target of rapamycin complex 1 (mTORC1) signaling pathway, which promotes cell metabolism and growth [30]; (ii) the caspase cascade, which leads to inflammation and cell death [31]; (iii) signaling pathways regulated by the nuclear factor kappa b (NF-κB), and (iv) signaling regulated by the mitogen-activated protein kinases (MAPK), which regulate inflammation, cell proliferation, and cell survival [32, 33]. We examined each of the above pathways for a role in infection-induced lipid droplet accumulation by treating infected macrophages with pathway-specific chemical inhibitors. Inhibitors of the MAPK and NF-κB pathways had no effect or induced only a modest increase (1.5 fold) in lipid droplet content of the infected cells (Fig 5A). The pronounced lipid droplet increase seen with cells from some donors is consistent with the notion that NF-κB mediates the lipolytic effects of TNFα [34]. In sharp contrast, the lipid droplet content of infected macrophages decreased by 50% when the cells were treated with inhibitors of mTORC1 and caspase 8 (an initiator caspase) [the 30% decrease observed with treatment with a caspase 3 (an effector caspase) inhibitor did not reach statistical significance (*p =* 0.09)] (Fig 5A). These results indicate that TAG accumulation in *M*. *tuberculosis*-infected macrophages requires functional mTORC1 signaling and activation of the caspase cascade.

The observed pro-lipogenic effects of the mTORC1 and caspase pathways in *M*. *tuberculosis* infection are consistent with functions previously reported for these pathways in the context of metabolic and neurodegenerative disorders and cancer [35–37]. For caspases, these include mitochondrial damage, activation of lipogenesis regulators, and blockage of autophagy. For mTORC1, the functions include induction of TAG biosynthesis and blockage of autophagy-dependent and -independent lipid degradation [38–44]. Reduced fatty acid utilization resulting from the metabolic reprograming induced by the mTORC1 pathway [45] is not involved in *M*. *tuberculosis*-driven TAG accumulation, since inhibiting HIF-1, which is the master transcriptional regulator of central metabolism in the mTORC1 pathway [46], does not decrease the lipid droplet content of infected macrophages (S5 Fig).

We also asked whether the TNFα, mTORC1, and caspase pathways are involved in human tuberculous granulomas. Analysis of transcriptomics data from tuberculous human lung tissue (GEO GSE20050) showed strong upregulation of these pathways in caseous granulomas relative to uninvolved lung (Fig 5B). Among the genes that contributed most to the upregulation of the corresponding pathway were genes encoding caspases (*CASP3* and *CASP8*), mTOR, and mTORC1 substrates 4E-BP1 and S6K1 (S4 Table). Even though signal transduction through these pathways is non-transcriptional, our gene expression data analysis clearly indicates that these pathways are perturbed during tuberculosis pathogenesis. Indeed, activation of mTORC1 in foam cells has been observed by histopathological analysis of a tuberculous human lung sample [47], which supports our in vitro data.

**Fig 6:**
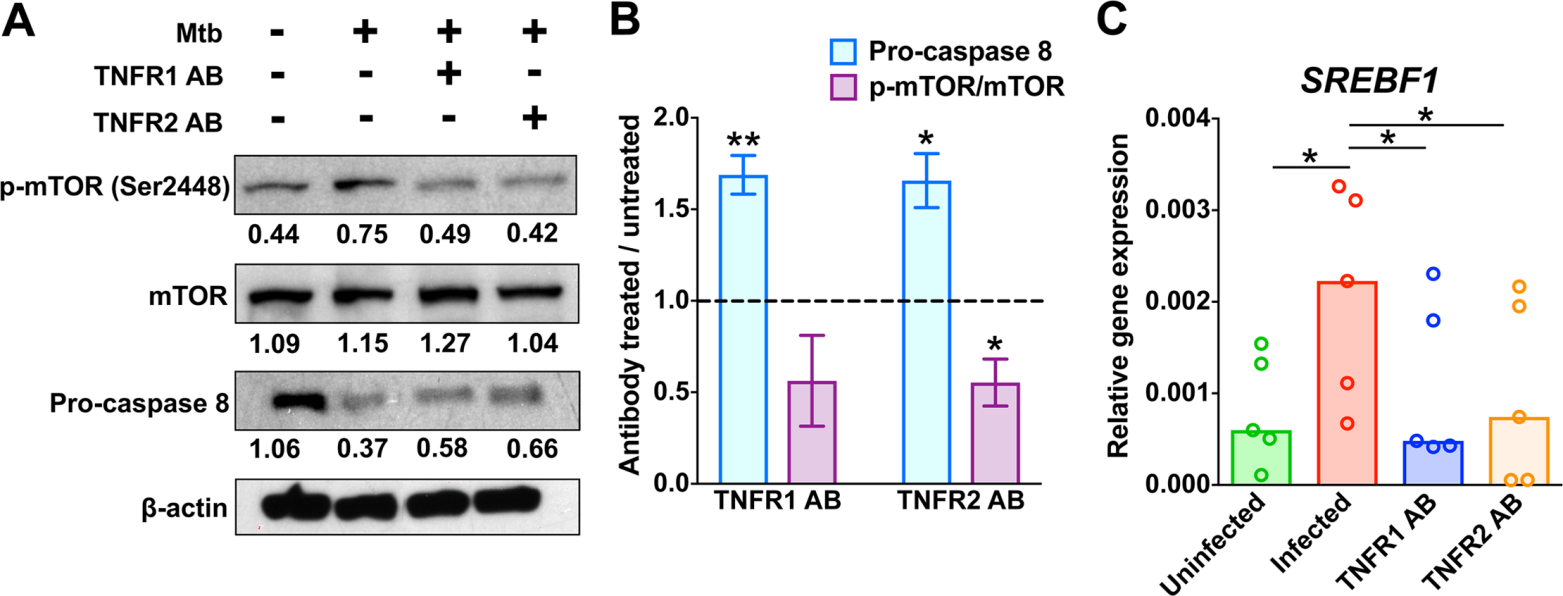
Effects of TNFR neutralizing antibodies on mTORC1 and caspase activity. MDM were treated with antibodies against TNFR1 or TNFR2 prior to infection. After 24 h of infection, whole cell lysates and RNA were obtained. (**A**) Analysis of phosphorylation state of mTORC1 and abundance of pro-caspase 8 by western blot. p-mTOR, band reacting with an antibody specific for mTOR phosphorylated at Ser-2448; mTOR, band reacting with an antibody recognizing total mTOR protein. Western blot bands were quantified by using ImageJ software. Numbers below each band indicate the intensity ratio of the test band relative to β-actin (loading control). Full-length blots are presented in S8 Fig. (**B**) Quantification of the western blot data in panel A. The graph shows the effect of TNFR antibody treatment (treated vs untreated) on the ratios of p-mTOR/mTOR and procaspase 8 abundance. The average and standard deviation for three donors is shown. (**C**) Gene expression analysis. Methods are described in the legend to Fig 3. The graph shows the median of five donors, with each dot representing one donor. Statistical significance (*p < 0.05, **p < 0.01) was assessed by one-sample (panel **B**) and paired (panel **C**) student t-tests. The comparisons in the paired tests are as indicated; the comparison in the one-sample student t-test was between treated and untreated cells. [It is noted that the decrease of 50% in the ratio of p-mTOR/mTOR observed with the anti-TNFR1 antibody treatment did not reach statistical significance (*p* = 0.09)]. Mtb: *M*. *tuberculosis*, TNFR1 AB: TNFR1 neutralizing antibodies, TNFR2 AB: TNFR2 neutralizing antibodies.

**Fig 7:**
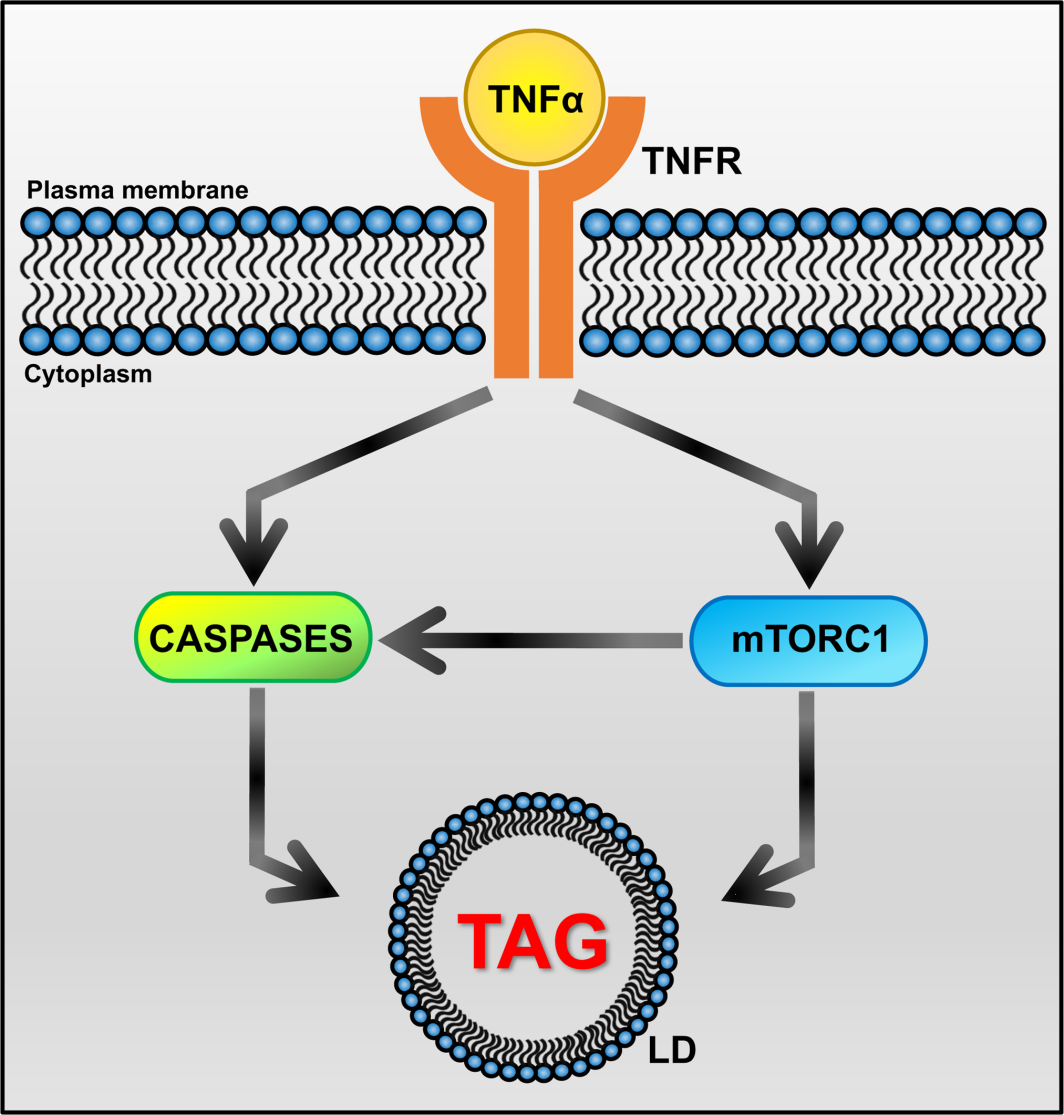
Key nodes of the signaling network involved in TAG accumulation in tuberculous macrophages. Infection of macrophages with *M*. *tuberculosis* induces production of TNFα, which has both autocrine and paracrine effects. TNFα binding to its surface receptors (TNFR1 and TNFR2) triggers the activation of intracellular caspase and mTORC1 pathways. Both mTORC1 and caspases directly interact with autophagy effectors to inhibit autophagic flux [42, 52, 109]. So does *M*. *tuberculosis* infection [110]. In addition, mTORC1-dependent block of autophagy can also activate the caspase cascade through accumulation of p62, which binds and activates caspase 8 [111]. When autophagy is blocked, lipid droplets are not degraded and accumulate in the cytoplasm. Our observation that treatment with TNFR neutralizing antibodies relieves the inhibition of autophagy by *M*. *tuberculosis* infection (S6 Fig) further supports inhibition of autophagy as a driver of lipid droplet accumulation in tuberculous macrophages. In addition, caspase activation also leads to mitochondrial dysfunction [43], which results in accumulation of lipid droplets due to reduced fatty acid utilization [39]. Effects on TAG biosynthesis may also be involved, since mTORC1 induces expression of *SREBF1* [112–114] and caspases activate SREBPs [40, 41]. Arrowhead: positive regulation. LD: lipid droplet.

We further explored mechanistic links between TNFα, mTORC1, and caspase pathways with respect to *M*. *tuberculosis*-induced TAG accumulation in macrophages. Activation of mTORC1 is associated with mTOR phosphorylation at residue Ser-2448 [48]. We observed that treating infected macrophages with TNFR neutralizing antibodies decreased mTOR phosphorylation at Ser-2448 (Fig 6A and 6B), as previously reported for cancer cells [49]; it also reduced expression of *SREBF1*, an mTORC1-activated gene (Fig 6C). Moreover, blocking TNFα signaling resulted in decreased caspase 8 activity in infected macrophages (Fig 6A and 6B), as expected from TNF signaling biology [50]. Thus, the three pathways we find associated with up-regulation of lipid droplet content are linked to each other in *M*. *tuberculosis-*infected macrophages (Fig 7).

## Discussion

The present report shows that the dominant type of storage lipid found in the necrotic and foam-cell-rich regions of tuberculous granulomas is TAG; moreover, the accumulation of this lipid class in *M*. *tuberculosis*-infected macrophages is driven by TNF receptor signaling, by downstream activation of the metabolic master regulator mTORC1, and by the caspase cascade. Both mTORC1 signaling and caspase activation regulate transcription factors (sterol regulatory element-binding proteins, SREBPs) involved in TAG biosynthesis [40, 41, 44, 51], and both block autophagy [42, 52], which is a major cellular lipid catabolic process [53]. Thus, increased TAG biosynthesis and/or inhibition of autophagy may be driving TAG accumulation in macrophages during *M*. *tuberculosis* infection. By characterizing the chemical nature of the dominant storage lipid in tuberculous foam cells and the underlying accumulation mechanisms, our work opens a new avenue of investigation into relationships between the human host and its deadliest pathogen [54].

Our work also demonstrates that the mechanisms underlying foam cell formation vary with the immunopathological context. In particular, tuberculous foam cells are characterized by the accumulation of TAG, in sharp contrast with the atherogenic foam cells, which are enriched in CE. Consequently, the mechanisms of macrophage lipid accumulation for these two major human diseases differ. During atherogenesis, macrophages develop into foam cells by inducing cholesterol-rich lipoprotein uptake, subverting cholesterol trafficking and reducing cholesterol efflux [2, 55]. In contrast, our findings strongly imply that, during *M*. *tuberculosis* infection, the development of macrophages into foam cells involves the activation of signaling pathways leading to intracellular TAG accumulation (Fig 7). Nevertheless, in both pathologies, the development of foam cells results in loss of macrophage functions associated with protection and/or inflammation resolution, such as reduced egress and defective efferocytosis in atheromas [55] and decreased phagocytosis and respiratory burst in tuberculous foam cells [7, 8]. Thus, regardless of the mechanism of foam cell biogenesis, their appearance is presumably a manifestation of maladaptive host responses.

Our work also contributes new insight into the effects of TNFα on lipid homeostasis. TNFα production is associated with dyslipidemia in many metabolic disorders (atherosclerosis, insulin resistance, obesity, and diabetes) and in cardiovascular disease (reviewed in [25, 26, 56–58]). However, most of the mechanistic studies exploring these associations have been performed with atherosclerosis, where TNFα promotes cholesterol accumulation in monocytes/macrophages, and atherogenesis [59–63]. Our discovery that, during *M*. *tuberculosis* infection, TNFα signaling contributes to the accumulation of a different class of storage lipid than reported previously with atherosclerosis establishes the novelty of the work.

Given the plasticity of macrophage responses [15] and the opposing effects that TNFα may have on neutral lipid accumulation (e.g., TNFα reduces the lipid content of adipocytes by lipolysis [34, 64, 65], and it induces liver steatosis by stimulating lipid biosynthesis in hepatocytes [66, 67]), our work does not exclude the possibility that TNFα also induces lipolysis in lesional macrophages during tuberculosis. Indeed, the lipid content of individual macrophages may vary with local TNFα levels, as determined by autocrine/paracrine circuitry. Additional determinants of the microenvironment (e.g., other cytokines, oxygen tension) and stochastic events may also contribute. Consequently, different macrophages in the same lesion may or may not exhibit the foamy phenotype, and granulomas may differ from one another within the same host.

By showing that the dominant neutral lipids in tuberculous foam cells are TAGs, our work may also further the understanding of the adaptive responses of tubercle bacilli during infection. It is recognized that *M*. *tuberculosis* responds to environmental cues by reprogramming its metabolism and halting growth [68, 69]. Host storage lipids may trigger or favor the pathogen’s metabolic reprogramming that is associated with mycobacterial persistence [7, 70]. Therefore, our finding that the lesional macrophages are TAG-rich increases the physiological relevance of the observation that, when macrophages are stimulated to accumulate TAGs in vitro, tubercle bacilli can utilize these host lipids to synthesize their own TAGs [71]. In turn, mycobacterial TAGs are presumed to be critical for survival of non-growing bacilli and their ability to resume growth during tuberculosis reactivation [72, 73]. Thus, TAG metabolism of both host and bacterial cells is central to tuberculosis pathogenesis.

Finding that TAG is the dominant storage lipid in tuberculous macrophages highlights a critical difference in the pathogenesis of the two major mycobacterial diseases. Unlike *M*. *tuberculosis*, *M*. *leprae*, the causative agent of leprosy, increases the levels of cholesteryl esters in macrophages [74]. Thus, infection with these two pathogenic mycobacteria results in radically different metabolic responses in the host macrophages. One explanation may lie with the different pattern recognition receptors engaged in lipid droplet biogenesis in the two infections: lipid droplet formation in *M*. *leprae*-infected cells requires both Toll-like receptor 2 (TLR2) and TLR6 [75], while *M*. *tuberculosis*-induced lipid droplets require TLR2, but not TLR-6 [76]. Further work is required to fully elucidate the mechanisms underlying the different pro-adipogenic mechanisms induced by these two mycobacteria. It is worth stressing that the dominant presence of TAGs in the *M*. *tuberculosis*-induced lipid droplets does not negate–and is fully compatible with–the critical role that free cholesterol has in *M*. *tuberculosis* pathogenesis. For example, altered intracellular trafficking of free cholesterol has been associated with endoplasmic reticulum stress [77], which also occurs in tuberculosis [78].

Our data may also contribute a mechanistic explanation for the association between tuberculosis and insulin resistance. In normal cells, binding of insulin to its receptor initiates a signaling cascade leading to increased glucose uptake into cells, storage of energy in the form of glycogen or triglycerides, mTORC1-mediated activation of protein synthesis, and various other effects [79]. Active mTORC1 inhibits the activity of factors associated with or immediately downstream of the insulin receptor, thereby blocking insulin signaling [18, 19]. This negative feedback loop contributes to metabolic homeostasis. It has been suggested that insulin desensitization associated with excess branched-chain amino acids may be secondary to insulin-independent mTORC1 hyperactivation [19], which disrupts homeostasis. Thus, it is tempting to propose that *M*. *tuberculosis* infection, which activates mTORC1 in an insulin-independent fashion ([45] and this work), may contribute to insulin desensitization during tuberculosis. Another contributing factor to insulin resistance may be production of TNFα during infection, since this cytokine inhibits insulin receptor signaling [20, 21]. Since insulin resistance is a powerful predictor of type 2 diabetes [80], the present work may reveal one of the mechanisms underlying the known links between tuberculosis and impaired glucose tolerance [81–83]—a condition that is tightly intertwined with insulin resistance [84]—and increased morbidity and severity of type 2 diabetes in tuberculous patients (http://www.who.int/tb/publications/tb-diabetes-framework/en/).

Finally, uncovering mechanisms of foam cell biogenesis during tuberculosis may have translational implications. The profiles of TAG species found in necrotizing tuberculous granulomas were conserved across host species and, presumably, across infecting *M*. *tuberculosis* strains (rabbits and marmosets were infected with two different, drug-sensitive strains, and the human lung donors were infected with multi-drug resistant bacteria). Strikingly, previous electrospray ionization mass spectrometry analysis of human tuberculous caseum [85] also revealed one of the dominant TAG species in Fig 2 (TAG 52:2), further supporting TAG conservation. Conserved TAG profiles imply utilization of conserved TAG biosynthetic enzymes (the acetyltransferases involved in TAG biosynthesis are expressed as diverse isoforms having high substrate specificity [86]). Thus, mRNAs and/or proteins involved in TAG biosynthesis may be exploited as novel biomarkers of progression to active tuberculosis. In addition, given the role of foam cells in tuberculosis pathogenesis, one might propose the therapeutic manipulation of factors affecting macrophage lipid content in tuberculosis, as already advocated for atherosclerosis and cardiovascular disease [2, 87, 88]. Since many of the factors we find associated with lipid droplet accumulation in tuberculous macrophages have been extensively studied in the context of cancer, cardiovascular, and metabolic diseases [89, 90], the possibility exists for re-purposing pharmacological compounds as host-directed therapy against tuberculosis. Such therapies may shorten the duration of anti-tuberculosis antibiotic regimens and/or help restrict the emergence of antibiotic resistance.

## Materials and methods

### Ethics statement

Animal studies were carried out in accordance with the Guide for the Care and Use of Laboratory Animals of the National Institutes of Health [91], with approval from the Institutional Animal Care and Use Committee of the New Jersey Medical School, Rutgers University, Newark, NJ (IACUC protocol number 16016D0319) and of the National Institute of Allergy and Infectious Diseases (NIAID), National Institutes of Health (NIH), Bethesda, MD (IACUC protocol number A-4149-01). Adults with pulmonary MDR-TB scheduled for elective lung resection surgery were asked to participate in the study “Pharmacokinetics of Standard First and Second Line anti-TB Drugs in the Lung and Lesions of Subjects Elected for Resection Surgery” (ClinicalTrials.gov NCT00816426). All subjects provided written informed consent. The institutional review boards of the NIAID, NIH and the Pusan National University Hospital, Asan Medical Center, and National Medical Center approved the study [92]. The procedures followed were in accordance with the ethical standards of the Helsinki Declaration [93].

### Bacterial strains

Human monocyte-derived macrophages (MDM) were infected in vitro with *M*. *tuberculosis* H_37_Rv. Rabbits were infected with *M*. *tuberculosis* HN878, and marmosets were infected with *M*. *tuberculosis* CDC1551. All *M*. *tuberculosis* strains, which are fully drug susceptible, belong to different lineages [94].

### Rabbit and marmoset infection

A total of ten rabbits were infected using a nose-only aerosol exposure system, and they were sacrificed at 12–16 weeks post-infection (four at PHRI and six at the NIAID) using established protocols [95, 96]. Nine marmosets were infected by the aerosol route, as described [23], and sacrificed at 6–8 weeks post-infection. Lung tissue samples containing lesions showing advanced necrosis (characterized by the presence of caseum) and areas of tissue free from visible lesions (uninvolved lung) were collected from all animals and frozen in liquid nitrogen vapor, as described [97].

### Tissue preparation for LC-MS

Tissue sections that were 25 μm thick were cut from gamma-irradiated lung tissue samples using a Microm HN505 N (Walldorf, Germany) and thaw-mounted onto 1.4 μm-thick Leica PET-Membrane FrameSlides (Herborn, Germany) for laser capture microdissection [92]. Tissue sections were immediately transferred into sealed containers and stored at -80°C. Adjacent 12 μm-thick tissue sections were thaw-mounted onto standard glass microscope slides for haematoxylin and eosin staining. Caseum and cellular areas in lesions were identified optically from a bright-field image scan and by comparison to the adjacently sectioned haematoxylin and eosin-stained tissue (S1 Fig). Equal areas (totaling 5 million μm^2^) of cellular (300 μM-wide regions surrounding the caseum) and caseous regions of necrotizing lung granulomas were dissected from three serial sections of lung tissue using a Leica LMD6500 system (Buffalo Grove, IL, USA) (S1 Fig). The mass of each pooled sample was calculated based on the surface area and tissue thickness of the pooled sections and an assumed tissue density of 1 g/ml, as previously described [98, 99]. Pooled dissected material was collected into 0.25 ml standard PCR tubes and immediately transferred to -80°C for storage.

Prior to analysis, samples were thawed at room temperature for 30 min, and 80 μl of extraction solution (Acetonitrile/Methanol (ACN/MeOH) 1:1) were added to each tube. The resulting suspension was sonicated for 5 min and centrifuged at 10,000 × *g* for 5 min at room temperature. 70 μl of the resulting supernatant were transferred into an equal volume of internal standard solution (d7-TAG, d7-CE, and d7-free cholesterol diluted in isopropyl alcohol, respectively at 200, 1000, and 1000 ng/ml) and mixed thoroughly. 100 μl of the resulting solution were utilized for LC-MS analysis.

Standards for quantification (1 to 50,000 μg/ml) were prepared from a stock solution prepared by diluting all free cholesterol, CE, and TAG standards to 100 μg/ml in ACN/MeOH 1:1 (containing d7-TAG, d7-CE, and d7-free cholesterol at 200, 1000, and 1000 ng/ml, respectively). A full list of standards used and their suppliers is shown in S1 Table. 50 μl of each standard were mixed with 50 μl of isopropyl alcohol (IPA) and 100 μl of the mixture were used for LC-MS analysis.

### LC-MS analysis

LC-MS analysis was performed on a Q Exactive high-resolution mass spectrometer (Thermo Fisher Scientific, Waltham, MA, USA) coupled to a Thermo Scientific Dionex UltiMate 3000 binary system. Chromatography was performed with a Kinetex C_18_ column (2.1 x 50 mm; particle size 1.7 μm, Phenomenex, Torrance, CA, USA) using reverse-phase gradient elution, ACN:H_2_O (60:40) and 10 mM ammonium acetate for mobile phase A, and IPA:ACN:MeOH (80:10:10) and 10 mM ammonium acetate for mobile phase B. A flow rate of 300 μl/min was used, with a gradient consisting of 20% B maintained for 0.5 min, followed by linear increase to 95% in 3.5 min, maintained for 2.2 min, and returned to the initial 20% B in 0.3 min. The column was equilibrated for 1.5 min before the subsequent injection, and the temperature of column and sample tray was maintained at 50 and 4°C, respectively.

Key MS parameters were as follows: spray voltage, 3.5 kV; capillary temperature, 320°C; HESI probe temperature, 400°C; S-lens RF level, 50. The sheath gas and auxiliary gas were set to 45 and 10 units, respectively. External mass calibration was performed before each sequence. Full scan was applied for all samples with a mass range of *m/z* 200–1500 at resolution power 70,000, AGC target 3e6 for a maximum IT of 100ms. Data-dependent MS/MS acquisition was performed with selected samples to acquire qualitative data validating lipid identities, with resolution power of 17,500, NCE 30 V, isolation window 1.0 *m/z*, intensity threshold 1.0e5, top 5 peaks and dynamic exclusion 3.0 sec.

Free cholesterol eluted at 5.5 min, TAG species eluted between 14 and 24 min, CE species eluted between 18 and 24 min. TAG species were quantified as NH4^+^ adducts and identified based upon retention time and reference to the Lipid Maps database (http://www.lipidmaps.org/tools/ms/lm_mass_form.php). CE species were quantified as Na^+^ adducts and identified based upon retention time and reference to the Lipid Maps database. Free cholesterol and CE identities were confirmed by the presence of the common cholesterol fragment [M+H-H_2_O]^+^ at *m/z* 369.356. An average calibration curve for TAG standards was generated, and TAG species in tissues and MDM were quantified utilizing the average curve (S7 Fig). After normalization to the internal standard, CE molecular species were quantified using the standard curve most similar in both chain length and unsaturation (S7 Fig) [100]. Free cholesterol signal was quantified after normalization to d7-cholesterol [M+H-H_2_O]^+^ signal (*m/z* 376.398).

### Confocal microscopy of lung tissue sections

Frozen lung tissue sections from *M*. *tuberculosis*-infected rabbits were fixed with 4% paraformaldehyde in 1× PBS for 10 min. After fixation, tissue sections were washed three times with 1× PBS, and permeabilized with 0.1% Triton X-100 in 1× PBS at room temperature for 5 min. Slides were then washed four times with 1× PBS, and stained with 0.3 μg/ml Nile red (Sigma-Aldrich, Saint Louis, MO, USA) at room temperature for 30 min. After washing four times with 1× PBS, tissue sections were mounted in ProLong Diamond Antifade Mountant containing DAPI (Invitrogen, Carlsbad, CA, USA), and cured at room temperature for 24 h. Images were captured by using a Leica TCS SP8 confocal microscope; a sequential image recording option was applied to avoid emission crosstalk between DAPI and Nile Red dyes. Image analysis was performed by using Leica LAS X software and ImageJ (https://imagej.nih.gov/ij/index.html).

### Human monocyte-derived macrophage infection and treatment

Human buffy coats were obtained from the New York Blood Center (Long Island City, NY, USA), and peripheral blood mononuclear cells (PBMC) were prepared by Ficoll density gradient centrifugation (Ficoll-Paque, GE Healthcare, Uppsala, Sweden) as described [101]. Isolated PBMC were washed and resuspended in serum-free RPMI-1640 medium (Corning, Manassas, VA, USA) supplemented with 4 mM L-glutamine (Corning, Manassas, VA, USA), seeded at a density of 1 × 10^7^ cells/ml in tissue culture multiwell plates or flasks, and incubated in humidified atmospheric air containing 5% CO_2_ at 37°C. After 4 h, non-adherent cells were removed by washing five times with 1× PBS and the adherent fraction was cultured over 7 days in culture medium (RPMI-1640 supplemented with 10% fetal bovine serum (Seradigm, Radnor, PA, USA) and 4 mM L-glutamine). At day 7, the medium was removed, and monocyte-derived macrophages were counted and infected with *M*. *tuberculosis*. The bacterial inoculum for infection was prepared by diluting a frozen bacterial stock in supplemented RPMI-1640 medium (as above) to obtain an MOI of 4 colony-forming units (cfu) per cell. Bacterial clumps were disrupted by vortexing with sterile 3-mm-diameter glass beads for 2 min; this suspension was used for MDM infection. Infected cells were treated with chemical inhibitors or with vehicle controls at the time of infection. When neutralizing antibodies were used, cells were treated with neutralizing antibodies for 1 h prior to infection. Inhibitor/antibody doses were selected based on available EC_50_ data and toxicity profiles with uninfected macrophages (MTS assay; CellTiter 96 Aqueous One Solution Cell Proliferation Assay Promega, Madison, WI, USA). Only inhibitor/antibody doses resulting in >90% cell viability were utilized. The following inhibitor/antibody concentrations were used: 0.078 μM R-7050 (TNFR inhibitor), 60nM A922500 (DGAT inhibitor) (Santa Cruz Biotechnology, Dallas, TX, USA), 0.4 nM rapamycin (mTORC1 inhibitor), 1.25 μM Z-IETD-FMK (caspase 8 inhibitor), 1.25 μM Z-DEVD-FMK (caspase 3 inhibitor), 7 nM QNZ (NF-κB inhibitor), 300 nM SB203580 (p38 MAPK inhibitor), 18.7 nM JNK-IN-8 (JNK MAPK inhibitor), 1.1 nM GDC-0994 (ERK MAPK inhibitor), 5 nM BAY 87–2243 (HIF-1α inhibitor) (Selleckchem, Houston, TX, USA), 1.5 μg/ml TNFR1 and 0.75 μg/ml TNFR2 neutralizing antibodies (Research and Diagnostic Systems, Minneapolis, MN, USA). After 24 h of incubation, macrophages were washed three times with 1× PBS and used for subsequent analysis.

### Lipid droplet quantification by imaging flow cytometry

For imaging flow cytometry, macrophages were detached from tissue culture plates by incubating with 5 mM EDTA in 1× PBS pH 8 for 30 min and gentle scraping, washed once with 1× PBS, and fixed with 4% paraformaldehyde in 1× PBS for 30 min at room temperature. Cells were then washed with 1× PBS containing 0.1% BSA (bovine serum albumin) (PBS-BSA), resuspended in 50 μl of PBS-BSA containing 5 μl of Fc receptor blocking solution, FcX (BioLegend, San Diego, CA), and incubated at room temperature for 5 min. After incubation, 50 μl of PBS-BSA containing 2.5 μl of CD11c APC (clone S-HCL-3; BD Biosciences) antibody were added to each tube, and samples were incubated at 4°C for 30 min. After washing with PBS-BSA, cells were stained with 0.3 μg/ml Bodipy 493/503 (Life Technologies, Carlsbad, CA) in 1× PBS for 15 min. For each experimental condition, data from 5,000–10,000 CD11c+ cells were acquired at the ImageStream^X^Mark II imaging flow cytometer (Amnis Corporation, Seattle, WA) using 60 × magnification. Image data were analyzed by IDEAS software version 6.0 (Amnis Corporation, Seattle, WA) after applying a compensation matrix and selecting the region of interest (lipid droplets) with the Spot Mask tool. Median fluorescence intensity per cell was extracted.

### Extraction of lipids from monocyte-derived macrophages

Macrophages were detached from tissue culture flasks, as described above, and washed once with 1× PBS. Cells obtained from each flask were counted by using a hemocytometer under a light microscope and centrifuged at 400 × *g*. Lipids were extracted from cell pellets with methanol, chloroform and water (2:1:2.5 by vol), dehydrated in a vacuum centrifuge at 4°C, and stored at -20°C.

### Whole cell lysates and immunoblotting

Western blotting was performed using the iBlot Dry Blotting System (Invitrogen, Carlsbad, CA, USA) according to the manufacturer’s instructions. A total of 2 × 10^6^ macrophages were lysed in 100 μl of RIPA lysis buffer (Santa Cruz Biotechnology, Dallas, TX, USA). Cell lysates were sterilized by filtration through 0.2 μm-pore filters and stored at -20°C. Equal amounts of protein (20–30 μg), determined by BCA Protein Assay (Thermo Fischer Scientific, Waltham, MA, USA), were resolved in each lane by SDS-PAGE on 7.5% polyacrylamide gels (Bio-Rad, Hercules, CA, USA). Separated proteins were transferred to PVDF membranes using the iBlot gel transfer stacks and the iBlot gel transfer device (Invitrogen, Carlsbad, CA, USA). Nonspecific binding sites were blocked by incubation of membranes with 5% blotting grade blocker (Bio-Rad, Hercules, CA, USA) in TBS-Tween buffer (20 mM Tris, 137 mM NaCl, 0.1% Tween-20) at room temperature for 2 h, followed by incubation with primary antibody in 5% (w/v) bovine serum albumin (Roche Diagnostics International AG, Rotkreuz, Switzerland) at 4°C. The following primary antibodies from Cell Signaling Technology (Danvers, MA, USA) were used at 1:1000 dilution: rabbit anti-p-mTOR (Ser2448, #2971), rabbit anti-mTOR (#2972), mouse anti-caspase 8 (#9746), and rabbit anti-β-actin (#4970). After overnight incubation, membranes were washed four times with TBS-Tween buffer and incubated with HRP-conjugated anti-rabbit (1:6000, #7074) or anti-mouse (1:10000, #7076) secondary antibodies (Cell Signaling Technology, Danvers, MA, USA) in TBS-Tween buffer containing 5% (w/v) milk at room temperature for 1 h. Membranes were washed four times with TBS-Tween buffer, and proteins were visualized using the LumiGLO chemiluminescent substrate solution (Cell Signaling Technology, Danvers, MA, USA), according to manufacturer’s instructions. Bands were quantified by using ImageJ software (https://imagej.nih.gov/ij/index.html).

### RNA isolation and RT-PCR

RNA was isolated by using TRI reagent (Molecular Research Center, Cincinnati, OH, USA) and following manufacturer’s instructions. Reverse transcription was performed by using random hexameric primers and ThermoScript Reverse Transcriptase (Invitrogen, Carlsbad, CA, USA). Enumeration of mRNAs was carried out by qPCR using gene-specific primers, molecular beacons and AmpliTaq Gold polymerase (Applied Biosystems, Foster City, CA, USA) in a Stratagene Mx4000 thermal cycler (Agilent Technologies, La Jolla, CA, USA). Nucleotide sequences of PCR primers and molecular beacons are listed in S5 Table. Gene expression was calculated using the 2 ^-ΔΔCt^ method [102] and normalized to the housekeeping *ACTB* gene.

### Transcriptome data analysis

Transcriptome data were obtained from Gene Expression Omnibus accession no. GSE20050 [85, 95]. One-sided *t* tests were performed to compare measurements of transcript levels between caseous human pulmonary tuberculous granulomas and normal lung parenchyma. To evaluate gene sets defined by Pathway Interaction Database (https://www.ncbi.nlm.nih.gov/Structure/biosystems/docs/biosystems_about.html), each pathway set was tested for extreme ranks of differential expression among all measured genes using the coincident extreme ranks in numerical observations test; multiple transcript measurements were combined as previously described [103]. The Benjamini–Hochberg method [104] was used to calculate the false discovery rate (FDR).

### Statistical analyses

Mann-Whitney and Wilcoxon signed-rank tests were used to calculate the statistical differences between lipid contents of animal lung tissues (Mann-Whitney test for comparisons between lung regions, and Wilcoxon signed-rank test for comparisons between TAG and CE within each lung region). To control for multiple comparisons, probability values of *p* < 0.025 were considered statistically significant based on the Bonferroni approach.

Paired and one-sample t-student tests were performed to assess the statistical significance of the data obtained from in vitro infected macrophages. Probability values of *p* < 0.05 were considered to represent statistically significant differences.

## Supporting information

S1 Fig Laser capture microdissection of tuberculous rabbit lung tissue. (**A**) Haematoxylin and eosin staining of tissue sections was used to identify the caseous (C) and cellular (CR) regions of granulomas and the uninvolved lung tissue (U) in adjacent tissue sections. (**B-C**) Bright-field images of rabbit lung tissue before (**B**) and after (**C**) laser capture microdissection (LCM).
(TIF)

S2 Fig Quantification of TAG and CE levels in human tuberculous necrotizing granulomas. (**A**) Haematoxylin and eosin staining of a tuberculous human lung tissue section identifies the caseous (C) and cellular (CR) regions of a large necrotic granuloma. (**B**) Higher magnification of the lesional area located inside the black box in panel **A.** The foam-cell-rich area at the interface between cellular region and caseum is indicated by the arrows. (**C**) Drug treatment of subjects. Lung tissue was removed by lobectomy from HIV-negative adults with pulmonary MDR-TB. Subjects were treated with a particular drug regimen for several weeks or months before surgery (background regimen) and with additional study drugs that were administered a few hours before surgery, at the indicated doses (ClinicalTrials.gov NCT00816426 and [93]). (**D**) TAG and CE levels in human tuberculous lung tissue. Areas of caseous and macrophage-rich cellular regions of lesions, and regions of uninvolved lung were sampled by laser capture microdissection. Lipids were extracted and TAG and CE species quantified by LC-MS. All measurements were expressed as micrograms of lipid per gram of tissue (μg/g). Two lesional areas (one per patient), and one uninvolved lung area (from one of the two patients) were analyzed. RIF: rifampicin, INH: isoniazid, PZA: pyrazinamide, MXF: moxifloxacin, KAN: kanamycin, AUG: amoxicillin/clavulanate, PAS: para-aminosalicylate, CS: cycloserine, CFZ:clofazimine, LZD: linezolid, TAG: triglycerides, CE: cholesteryl esters.
(TIF)

S3 Fig TAG species profile in *M*. *tuberculosis*-infected macrophages. TAG species in infected and uninfected MDM were quantified by LC-MS. Each line of the graph represents one sample: MDM were obtained from 3 different donors (one uninfected sample and one infected sample per donor). The TAG species profile is similar to that obtained from animal and human tuberculous granulomas, with a slightly higher abundance of long-chain triglycerides.
(TIF)

S4 Fig Lipid droplet studies in THP-1 cells. These studies were performed with human macrophage-like cell lines rather than primary macrophages due to the cell numbers needed for lipid droplet isolation and analysis (5 × 10^7^ cells). (**A**) Lipid droplet visualization in THP-1 cells. Representative image of a THP-1 cell stained with Bodipy 493/503 and visualized by imaging flow cytometry (60× magnification), as described in Fig 3B. (**B**) Isolation of lipid droplets from THP-1 cells by density gradient centrifugation. Cells were lysed, nuclei were removed by low-speed centrifugation, and the density of the post-nuclear supernatant was adjusted with sucrose prior to flotation of the lipid droplets through a single discontinuous sucrose gradient, as described [115]. LD, floating opaque lipid droplet layer in the ultracentrifuge tube. (**C**) Isolated lipid droplets stained with Bodipy 493/503 and imaged by fluorescence microscopy (100× magnification). (**D**-**E**) Extracted ion chromatograms for free cholesterol signal at *m/z* 369.358 (**D**) and TAG (52:2) at *m/z* 876.802 (**E**). Free cholesterol is detected in the whole cell lysate extract (**D**, upper panel) but not in the isolated lipid droplet extract (**D**, lower panel); in contrast, TAG is detected in both extracts (**E**). (**F**) Absolute quantification of TAG and CE content by LC-MS. Infection of THP-1 cells with *M*. *tuberculosis* increased TAG content; CE was below the limit of quantification, in agreement with the results obtained with primary human macrophages. (**G**) Measurements of TAG and free cholesterol content by biochemical assays. Intracellular levels of TAG and free cholesterol were measured by using fluorometric assays (Total Cholesterol and Cholesteryl Ester Colorimetric/Fluorometric Assay Kit and Triglyceride Quantification Colorimetric/Fluorometric Kit, BioVision Inc., Milpitas, CA, USA). Infection of THP-1 cells with *M*. *tuberculosis* increased TAG but not free cholesterol content. (**H**) Effect of BM 15766 on lipid droplet content. THP-1 cells were infected with *M*. *tuberculosis* and treated with either DMSO (vehicle control) or BM 15766 (Santa Cruz Biotechnology, Dallas, TX, USA), a chemical inhibitor of the 7-dehydrocholesterol reductase, the enzyme catalyzing the last step of *de novo* cholesterol synthesis. After treatment, cells were stained with Bodipy 493/503 and visualized by imaging flow cytometry. (**-**), treatment with DMSO; (**+**) treatment with BM 15766. Treatment with BM 15766 had no effect on lipid droplet levels of infected THP-1 cells. In **F**, **G**, and **H**, average and standard deviation of triplicate experiments are shown. Statistical significance was evaluated by paired student t-test (*p < 0.05, **p < 0.01, ***p < 0.001). CHO: free cholesterol, TAG: triglycerides, UN: uninfected, INF: infected.
(TIF)

S5 Fig Effect of a HIF-1 inhibitor on lipid droplet content of *M*. *tuberculosis-*infected macrophages. MDM were infected with *M*. *tuberculosis* and treated with either DMSO (vehicle control) or BAY87-2243 (HIF-1α inhibitor). Lipid droplet content was quantified and results expressed as described in Fig 4.
(TIF)

S6 Fig Effect of blocking TNFR signaling on autophagy in *M*. *tuberculosis*-infected macrophages. MDM were pre-treated with antibodies against TNFR1 or TNFR2 prior to infection. After 24 h of infection, whole cell lysates were obtained. The abundance of p62 protein was analyzed by western blot. We used p62 levels as an autophagy marker, since this protein accumulates when autophagosome-lysosome fusion is inhibited [116]. Thus, decreased p62 levels in treated infected macrophages indicate resumption of autophagic flux. Western blot bands were quantified by using ImageJ software. The left panel shows a representative image of western blots. Numbers below each band indicate the intensity ratio of the test band relative to the β-actin band (loading control). Full-length blots are presented in S8 Fig. The right panel shows ratios of p62 abundance in TNFR antibody-treated cells relative to untreated controls for three donors. Means and standard deviations are shown.
(TIF)

S7 Fig Representative calibration curves used to quantify TAG and CE levels by LC-MS. An average curve was prepared for TAG from response ratios of seven representative TAG species. To quantify CE species, individual standard curves for four reference CE species were generated. The standard CE curve for the species closest to the analyte (as determined by the number of double bonds and carbon chain length) was used.
(TIF)

S8 Fig Full-length images of blots presented in Fig 6A and S6 Fig. Boxes shows the cropped blot regions presented in the figures. Numbers on the left side indicate the position of the molecular weight marker. U: uninfected, I: infected, R1AB: TNFR1 neutralizing antibodies, R2AB: TNFR2 neutralizing antibodies.
(TIF)

S1 Table List of lipid standards used for LC-MS quantification. (DOCX)

S2 Table LC-MS quantitative data by TAG species. (XLSX)

S3 Table LC-MS quantitative data by CE species. (XLSX)

S4 Table List of the most highly differentially expressed genes of pathways highlighted in Fig 5B. For each pathway, the genes in the top 10% of increased expression in caseous granuloma compared to lung parenchyma were tabulated. Statistics for the pathway analysis results and differential expression of gene measurements are shown for each pathway. The methods used were described in [103].
(XLSX)

S5 Table Primers and probes used in the study. (DOCX)

S1 Video Three-dimensional reconstruction of tuberculous rabbit lung tissue sections. Confocal Z-stacks of the lesional cellular region immediately surrounding the caseum were reconstructed into 3-D animations. Nile red-stained lipid droplets (red) are visible around the DAPI-stained cellular nuclei (blue).
(MOV)
