# Supplementary figures and images for: Storage lipid studies in tuberculosis reveal that foam cell biogenesis is disease-specific
Source: PLoS Pathog. 2018 Aug 30;14(8):e1007223. doi: 10.1371/journal.ppat.1007223 (PMC6117085; doi:10.1371/journal.ppat.1007223)

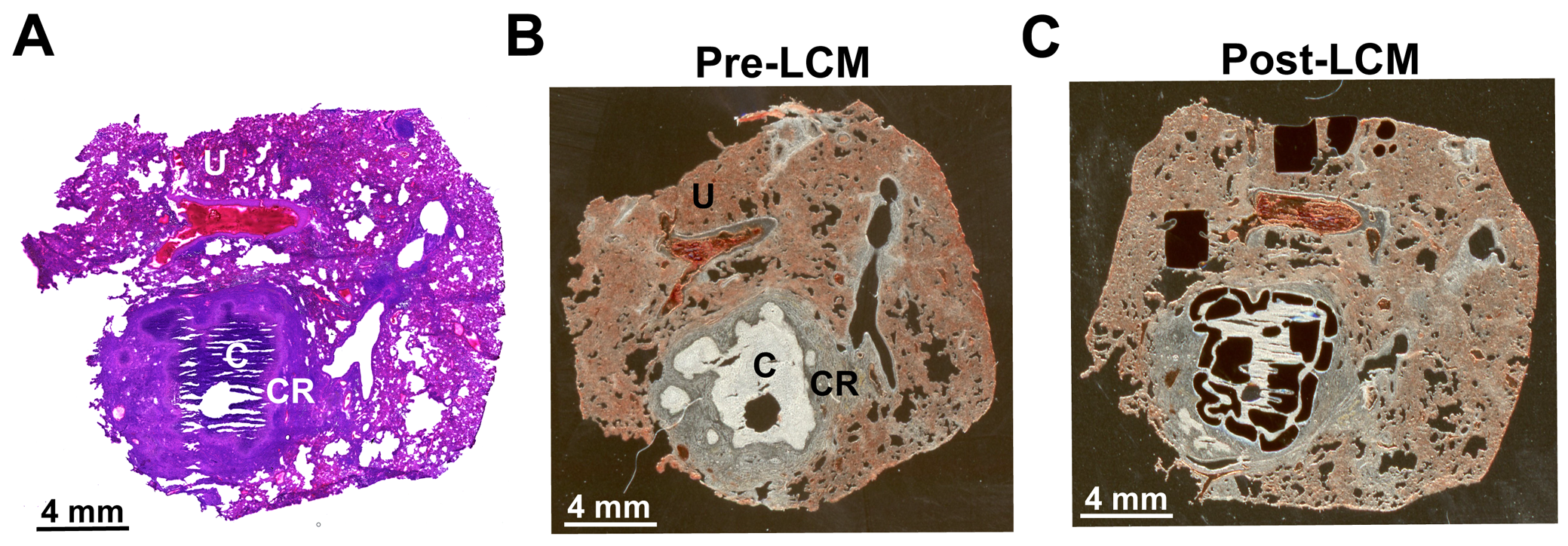

Supplement: S1 Fig — (A) Haematoxylin and eosin staining of tissue sections was used to identify the caseous (C) and cellular (CR) regions of granulomas and the uninvolved lung tissue (U) in adjacent tissue sections. (B-C) Bright-field images of rabbit lung tissue before (B) and after (C) laser capture microdissection (LCM). (TIF) [file ppat.1007223.s001.tif]

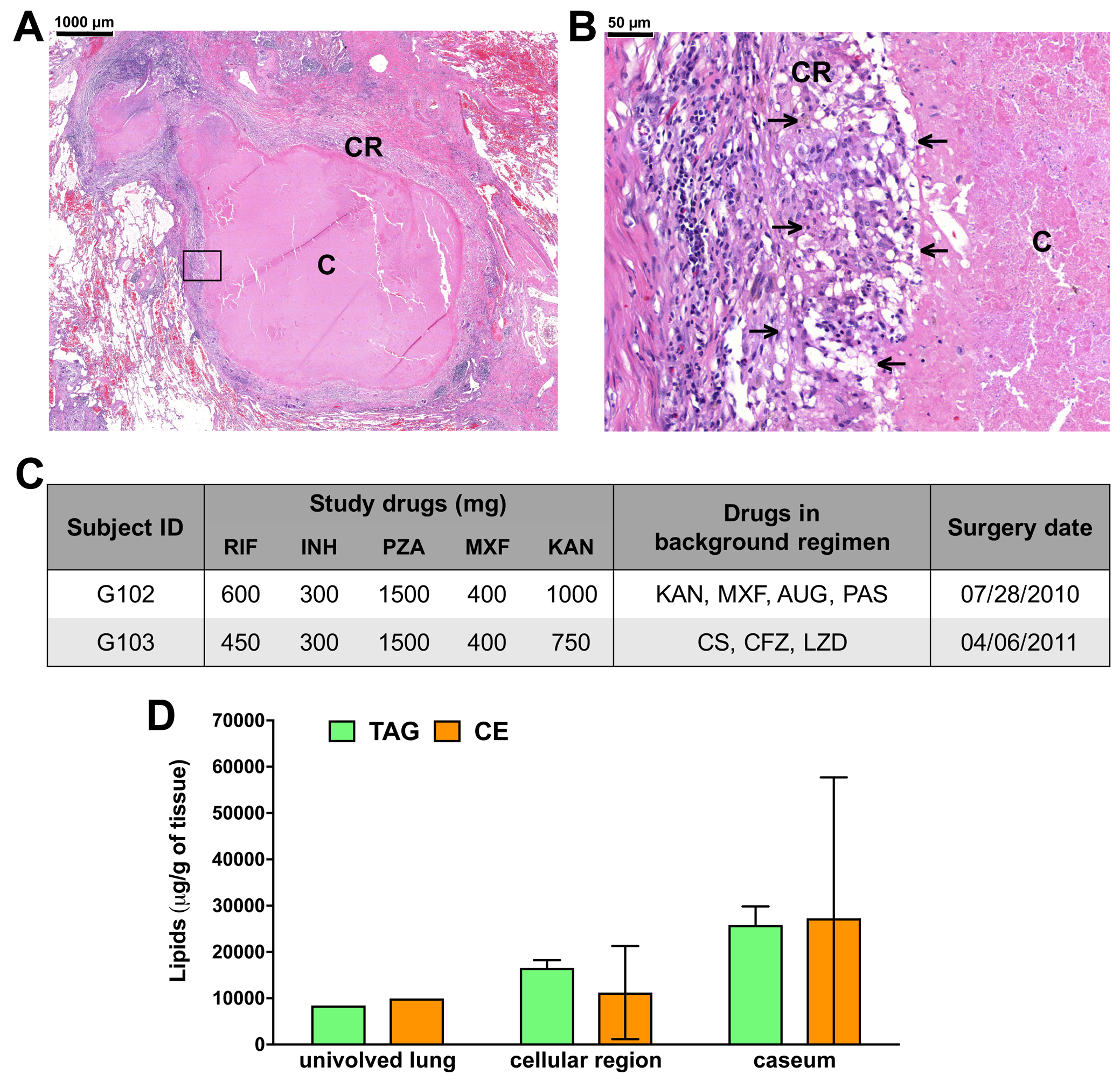

Supplement: S2 Fig — (A) Haematoxylin and eosin staining of a tuberculous human lung tissue section identifies the caseous (C) and cellular (CR) regions of a large necrotic granuloma. (B) Higher magnification of the lesional area located inside the black box in panel A. The foam-cell-rich area at the interface between cellular region and caseum is indicated by the arrows. (C) Drug treatment of subjects. Lung tissue was removed by lobectomy from HIV-negative adults with pulmonary MDR-TB. Subjects were treated with a particular drug regimen for several weeks or months before surgery (background regimen) and with additional study drugs that were administered a few hours before surgery, at the indicated doses (ClinicalTrials.gov NCT00816426 and [93]). (D) TAG and CE levels in human tuberculous lung tissue. Areas of caseous and macrophage-rich cellular regions of lesions, and regions of uninvolved lung were sampled by laser capture microdissection. Lipids were extracted and TAG and CE species quantified by LC-MS. All measurements were expressed as micrograms of lipid per gram of tissue (μg/g). Two lesional areas (one per patient), and one uninvolved lung area (from one of the two patients) were analyzed. RIF: rifampicin, INH: isoniazid, PZA: pyrazinamide, MXF: moxifloxacin, KAN: kanamycin, AUG: amoxicillin/clavulanate, PAS: para-aminosalicylate, CS: cycloserine, CFZ:clofazimine, LZD: linezolid, TAG: triglycerides, CE: cholesteryl esters. (TIF) [file ppat.1007223.s002.tif]

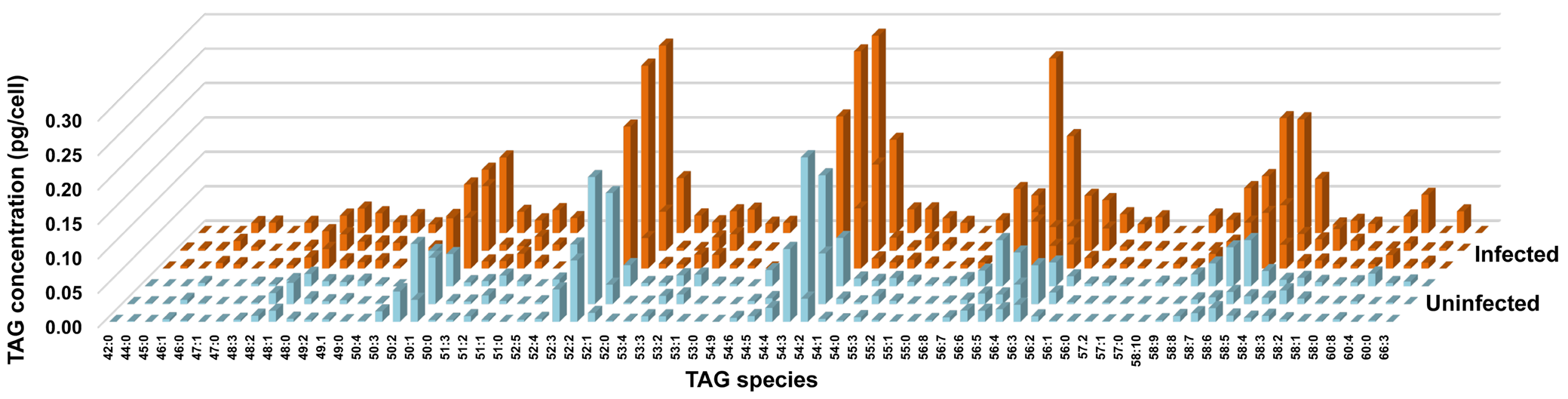

Supplement: S3 Fig — TAG species in infected and uninfected MDM were quantified by LC-MS. Each line of the graph represents one sample: MDM were obtained from 3 different donors (one uninfected sample and one infected sample per donor). The TAG species profile is similar to that obtained from animal and human tuberculous granulomas, with a slightly higher abundance of long-chain triglycerides. (TIF) [file ppat.1007223.s003.tif]

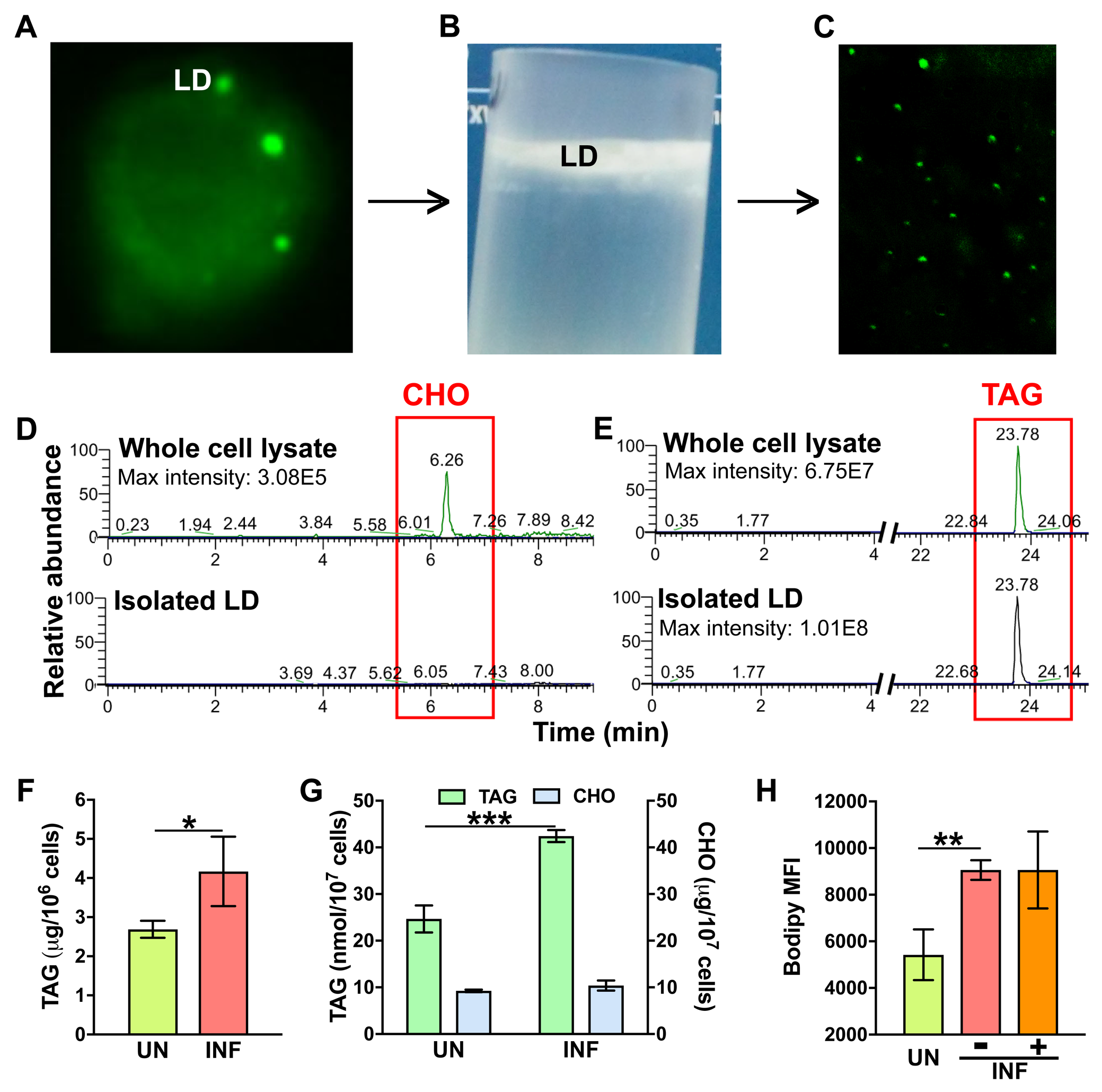

Supplement: S4 Fig — These studies were performed with human macrophage-like cell lines rather than primary macrophages due to the cell numbers needed for lipid droplet isolation and analysis (5 × 10^7^ cells). (A) Lipid droplet visualization in THP-1 cells. Representative image of a THP-1 cell stained with Bodipy 493/503 and visualized by imaging flow cytometry (60× magnification), as described in Fig 3B. (B) Isolation of lipid droplets from THP-1 cells by density gradient centrifugation. Cells were lysed, nuclei were removed by low-speed centrifugation, and the density of the post-nuclear supernatant was adjusted with sucrose prior to flotation of the lipid droplets through a single discontinuous sucrose gradient, as described [115]. LD, floating opaque lipid droplet layer in the ultracentrifuge tube. (C) Isolated lipid droplets stained with Bodipy 493/503 and imaged by fluorescence microscopy (100× magnification). (D-E) Extracted ion chromatograms for free cholesterol signal at m/z 369.358 (D) and TAG (52:2) at m/z 876.802 (E). Free cholesterol is detected in the whole cell lysate extract (D, upper panel) but not in the isolated lipid droplet extract (D, lower panel); in contrast, TAG is detected in both extracts (E). (F) Absolute quantification of TAG and CE content by LC-MS. Infection of THP-1 cells with M. tuberculosis increased TAG content; CE was below the limit of quantification, in agreement with the results obtained with primary human macrophages. (G) Measurements of TAG and free cholesterol content by biochemical assays. Intracellular levels of TAG and free cholesterol were measured by using fluorometric assays (Total Cholesterol and Cholesteryl Ester Colorimetric/Fluorometric Assay Kit and Triglyceride Quantification Colorimetric/Fluorometric Kit, BioVision Inc., Milpitas, CA, USA). Infection of THP-1 cells with M. tuberculosis increased TAG but not free cholesterol content. (H) Effect of BM 15766 on lipid droplet content. THP-1 cells were infected with M. tuberculosis and tr [file ppat.1007223.s004.tif]

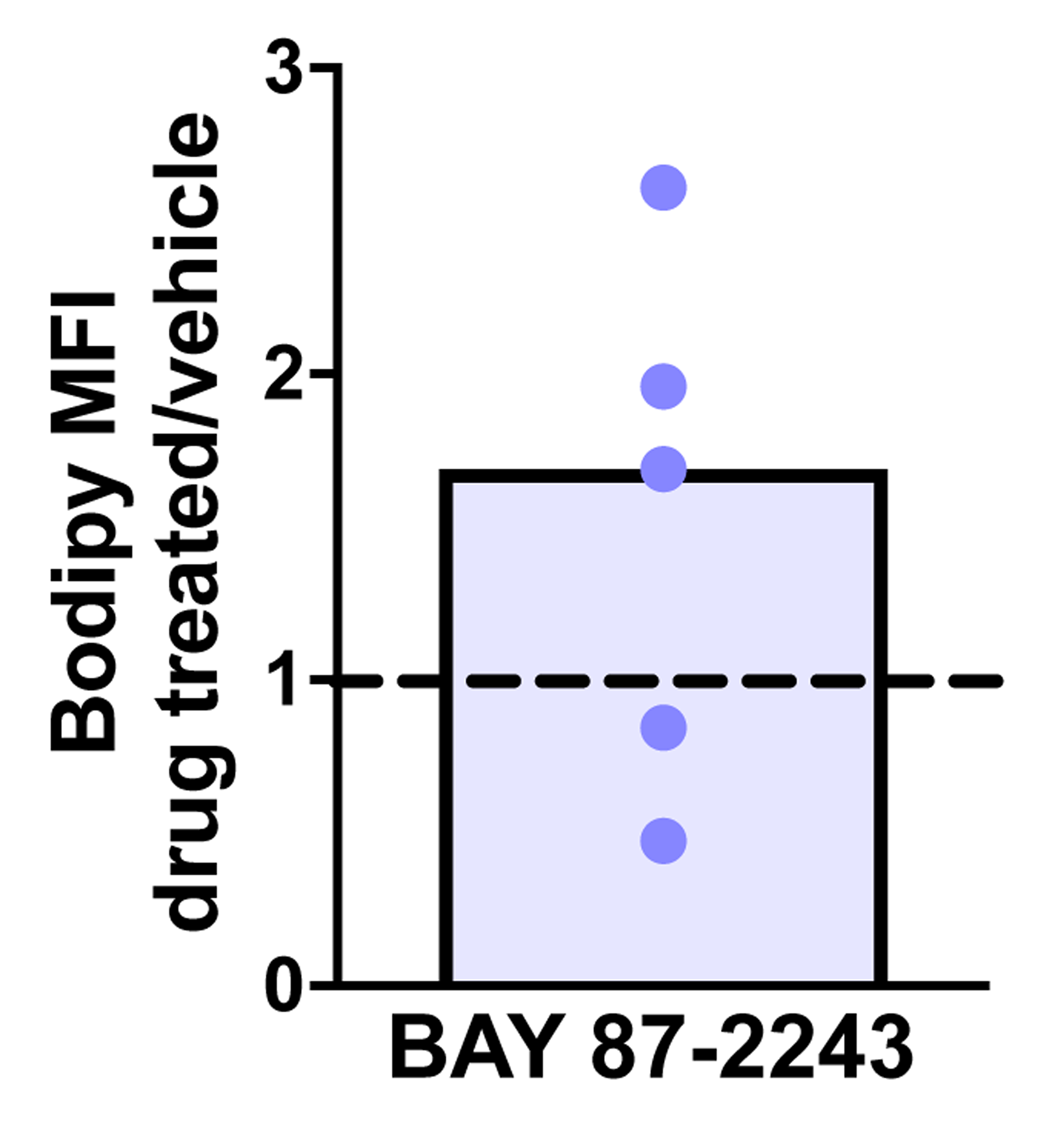

Supplement: S5 Fig — MDM were infected with M. tuberculosis and treated with either DMSO (vehicle control) or BAY87-2243 (HIF-1α inhibitor). Lipid droplet content was quantified and results expressed as described in Fig 4. (TIF) [file ppat.1007223.s005.tif]

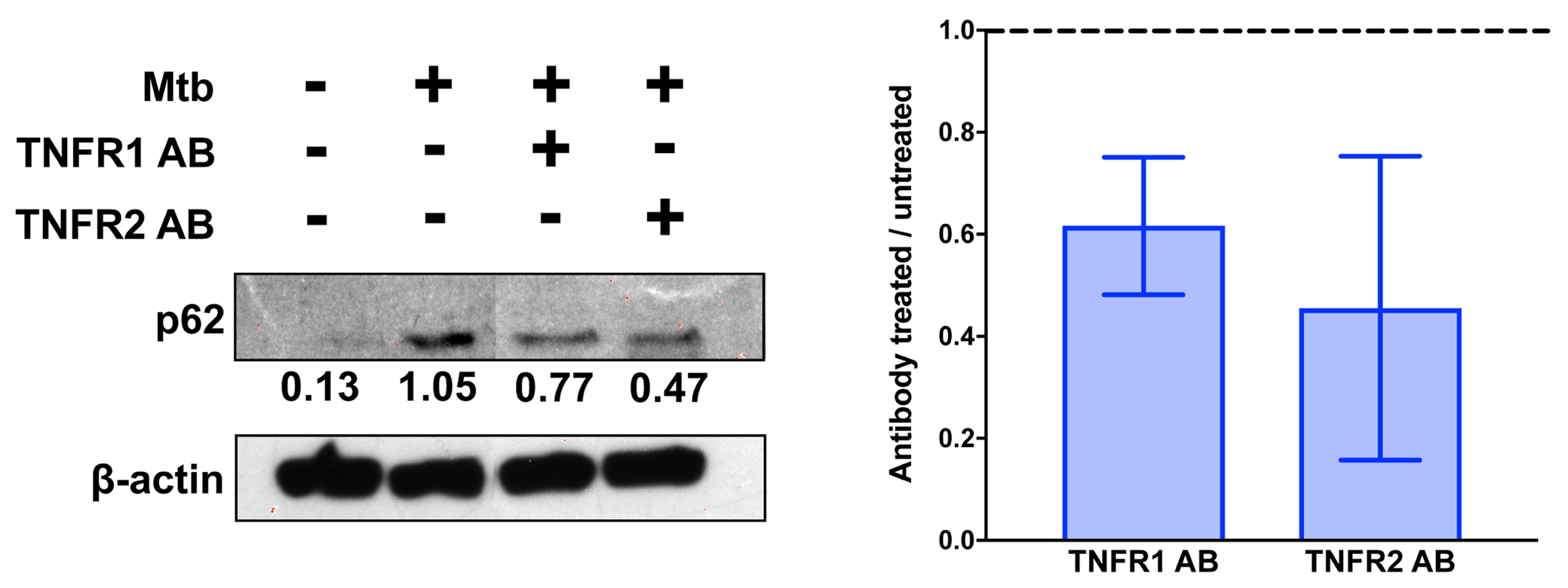

Supplement: S6 Fig — MDM were pre-treated with antibodies against TNFR1 or TNFR2 prior to infection. After 24 h of infection, whole cell lysates were obtained. The abundance of p62 protein was analyzed by western blot. We used p62 levels as an autophagy marker, since this protein accumulates when autophagosome-lysosome fusion is inhibited [116]. Thus, decreased p62 levels in treated infected macrophages indicate resumption of autophagic flux. Western blot bands were quantified by using ImageJ software. The left panel shows a representative image of western blots. Numbers below each band indicate the intensity ratio of the test band relative to the β-actin band (loading control). Full-length blots are presented in S8 Fig. The right panel shows ratios of p62 abundance in TNFR antibody-treated cells relative to untreated controls for three donors. Means and standard deviations are shown. (TIF) [file ppat.1007223.s006.tif]

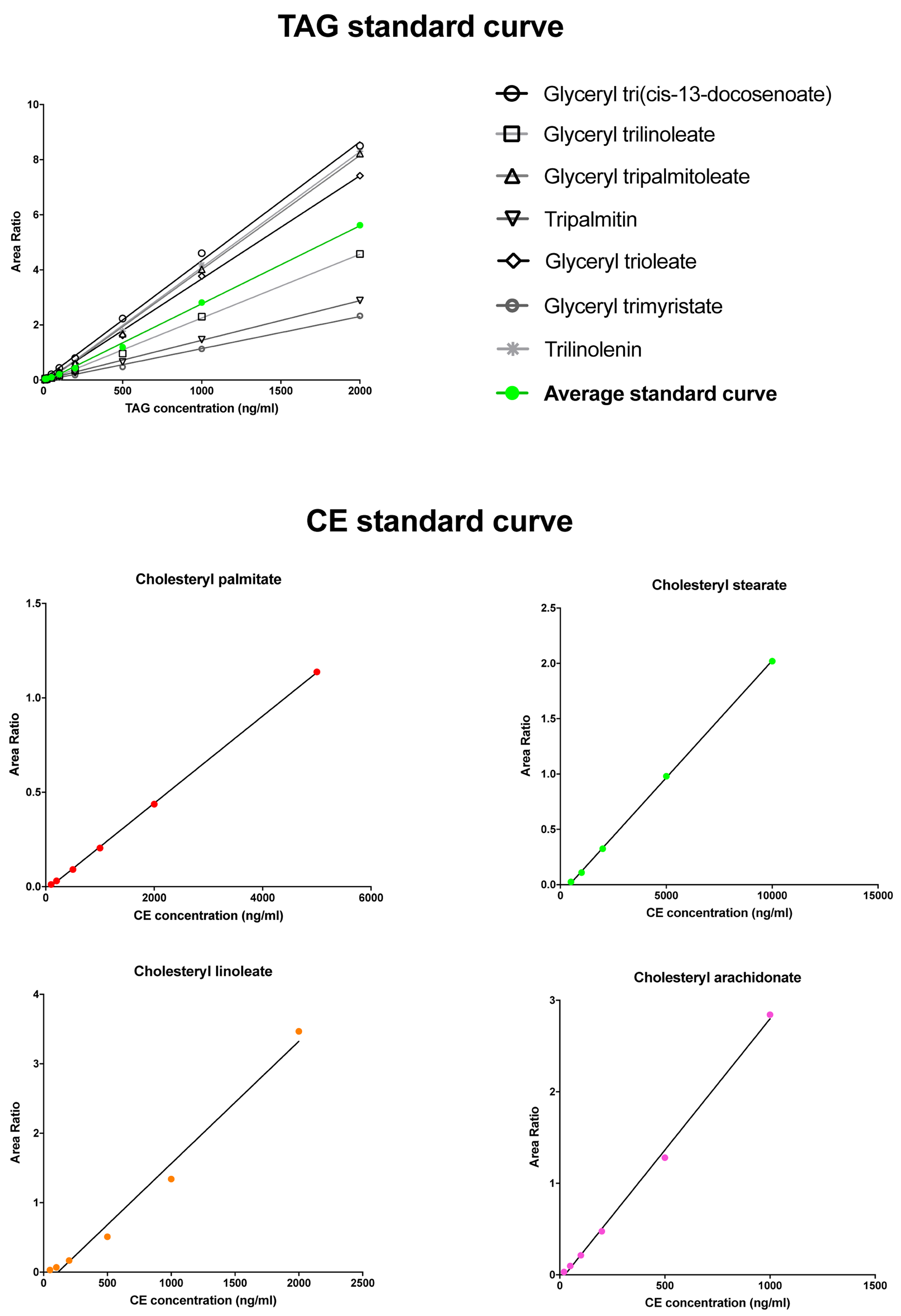

Supplement: S7 Fig — An average curve was prepared for TAG from response ratios of seven representative TAG species. To quantify CE species, individual standard curves for four reference CE species were generated. The standard CE curve for the species closest to the analyte (as determined by the number of double bonds and carbon chain length) was used. (TIF) [file ppat.1007223.s007.tif]

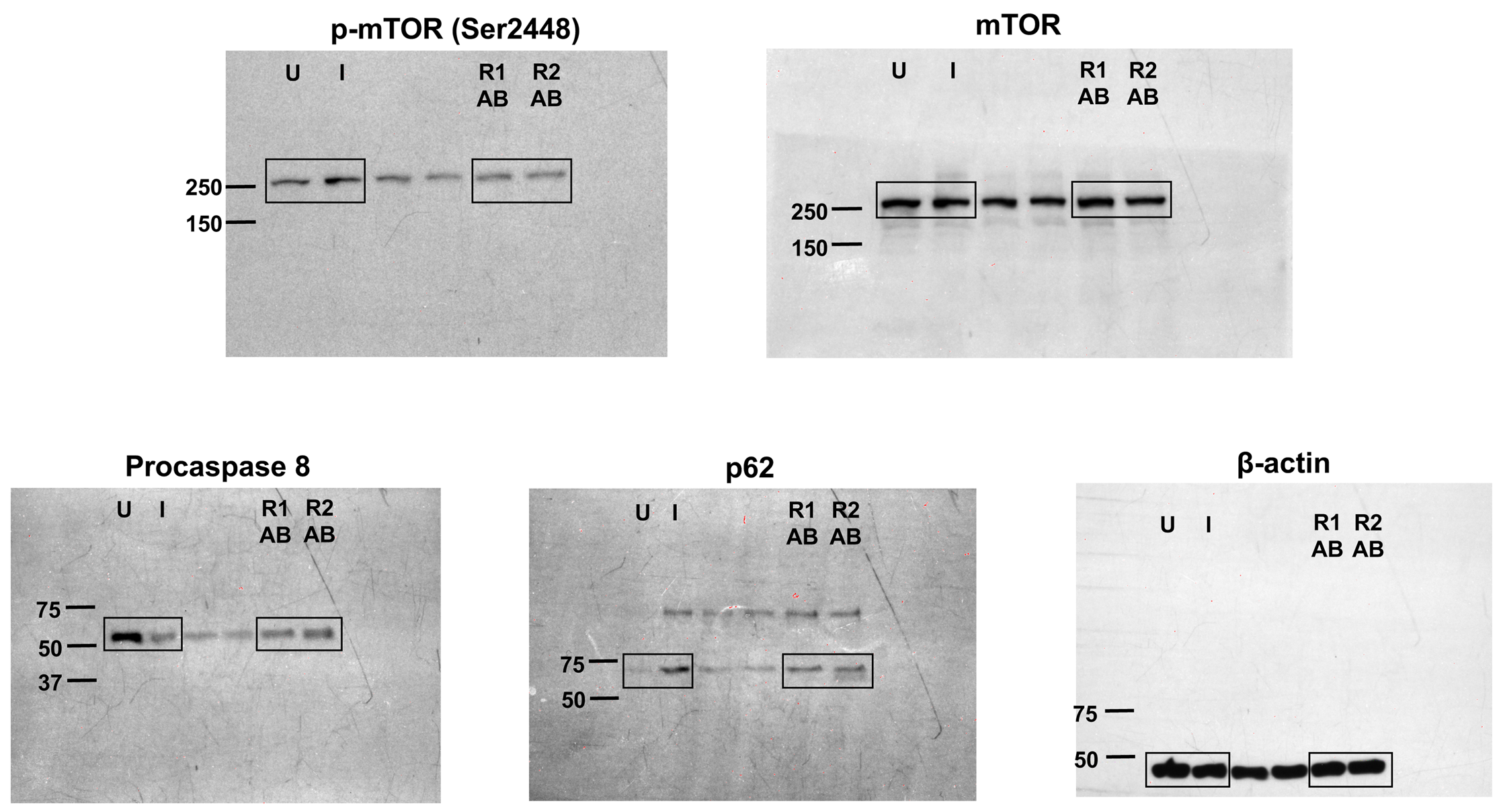

Supplement: S8 Fig — Boxes shows the cropped blot regions presented in the figures. Numbers on the left side indicate the position of the molecular weight marker. U: uninfected, I: infected, R1AB: TNFR1 neutralizing antibodies, R2AB: TNFR2 neutralizing antibodies. (TIF) [file ppat.1007223.s008.tif]
