# Supplementary material for: Storage lipid studies in tuberculosis reveal that foam cell biogenesis is disease-specific
Source: PLoS Pathog. 2018 Aug 30;14(8):e1007223. doi: 10.1371/journal.ppat.1007223 (PMC6117085; doi:10.1371/journal.ppat.1007223)
Supplement: S1 Table — (DOCX) [file ppat.1007223.s009.docx]

| **Lipid** | **Supplier** |
| --- | --- |
| Cholesterol | Sigma-Aldrich |
| D7-Cholesterol | Avanti Polar Lipids |
| Cholesteryl palmitate | Sigma-Aldrich |
| Cholesteryl stearate | Sigma-Aldrich |
| Cholesteryl linoleate | Sigma-Aldrich |
| Cholesteryl arachidonate | Sigma-Aldrich |
| Cholesteryl palmitate-d7 | Avanti polar lipids |
| Trilinolenin | Sigma-Aldrich (Triglyceride Kit TRI19-1KT) |
| Glyceryl trimyristate | Sigma-Aldrich (Triglyceride Kit TRI19-1KT) |
| Glyceryl tri(cis-13-docosenoate) | Sigma-Aldrich (Triglyceride Kit TRI19-1KT) |
| Glyceryl tripalmitoleate | Sigma-Aldrich (Triglyceride Kit TRI19-1KT) |
| Glyceryl trilinoleate | Sigma-Aldrich (Triglyceride Kit TRI19-1KT) |
| Tripalmitin | Sigma-Aldrich (Triglyceride Kit TRI19-1KT) |
| Glyceryl trioleate | Sigma-Aldrich (Triglyceride Kit TRI19-1KT) |
| 1,3-dipentadecanoyl-2-oleyol(d7)-glycerol | Avanti Polar Lipids |

**S1 Table**. **List of lipid standards used for LC-MS quantification.**
