# Supplementary material for: Storage lipid studies in tuberculosis reveal that foam cell biogenesis is disease-specific
Source: PLoS Pathog. 2018 Aug 30;14(8):e1007223. doi: 10.1371/journal.ppat.1007223 (PMC6117085; doi:10.1371/journal.ppat.1007223)
Supplement: S5 Table — (DOCX) [file ppat.1007223.s013.docx]

**S5 Table. Primers and probes used in the study.**

| **Gene** | **Forward primer (5’-3’)** | **Reverse Primer (5’-3’)** | **Molecular Beacon (5’-3’)** |
| --- | --- | --- | --- |
| *SREBF1* | CATTGAGCTCAAGGATCTGGT | TAGGTTCTCCTGCTTGAGTTTC | ACCCGCTTCGCTTTCTGCAACACAGCAACCGCGGGT |
| *LPIN1* | CCCAACACAGAACCCTTT | GACACTCCTACTTGCTTGTAT | ACCCGCTTGGAAACCGACCAGCTGATGTGTGCGGGT |
| *ACTB* | ACAGAGCCTCGCCTTTGC | ATGCCGGAGCCGTTGTC | ACGGGCATGGATGATGATATCGCCGCGCTCGCCCGT |
